# Dose–response analysis between weight-adjusted waist index and all-cause and cardiovascular mortality: a systematic review and meta-analysis

**DOI:** 10.3389/fnut.2026.1766501

**Published:** 2026-06-24

**Authors:** Pengyu Yang, Neng Huang, Jing Ye, Mingxin He, Yao Wu, Dan Liu, Hua Zhang, Ping Lei Chui, Zuoyan Liu

**Affiliations:** 1 Department of Rehabilitation Medicine, West China School of Nursing, Sichuan University, Chengdu, China; 2 Department of Critical Care Medicine, Peking University Shenzhen Hospital, Shenzhen, China; 3 Department of Nursing, Leshan Vocational and Technical College, Leshan, China; 4 Department of Psychiatry, Ziyang Psychosis Hospital, Ziyang, China; 5 School of Nursing, Hainan Medical University, Haikou, China; 6 Department of Nursing Science, Faculty of Medicine, Universiti Malaya, Kuala Lumpur, Malaysia

**Keywords:** all-cause mortality, cardiovascular mortality, dose–response, meta-analysis, weight-adjusted waist index, WWI

## Abstract

**Background:**

The weight-adjusted waist index (WWI), calculated as waist circumference divided by the square root of body weight (cm/ √ kg), has been proposed as an anthropometric index reflecting central adiposity relative to body weight. However, its association with all-cause and cardiovascular (CV) mortality remains incompletely characterized.

**Methods:**

We systematically searched PubMed, Embase, Web of Science, Scopus, and Medline from inception to April, 2026, for cohort studies evaluating WWI and all-cause or CV mortality. Hazard ratios (HRs) and 95% confidence intervals (CIs) were pooled using random-effects models. To avoid pseudo-replication from overlapping public database-derived cohorts, particularly NHANES, the primary analysis was based on non-overlapping cohorts. Risk of bias was assessed using the Newcastle–Ottawa Scale, and certainty of evidence was evaluated using the GRADE framework.

**Results:**

A total of 47 studies were included. In the primary non-overlapping analyses, each 1 cm/ √ kg increment in WWI was associated with a higher risk of all-cause mortality (HR = 1.21, 95% CI: 1.14–1.28; *I*^2^ = 83.5%) and CV mortality (HR = 1.27, 95% CI: 1.18–1.35; *I*^2^ = 60.8%). Comparing the highest with the lowest WWI categories yielded pooled HRs of 1.40 (95% CI: 1.22–1.61) for all-cause mortality and 1.54 (95% CI: 1.22–1.94) for CV mortality. Dose–response meta-analyses suggested a positive association compatible with a linear pattern. Although most included studies were of moderate to high methodological quality based on the NOS, the overall GRADE certainty of evidence was rated low for all-cause mortality and moderate for CV mortality, due to substantial between-study heterogeneity and potential publication bias.

**Conclusion:**

Higher WWI is positively associated with an increased risk of all-cause and CV mortality. WWI may serve as a useful epidemiological marker of adiposity-related mortality risk. However, given the observational nature of the evidence and substantial heterogeneity, further research with formal predictive performance evaluations is required to determine whether WWI improves individual-level risk prediction beyond traditional anthropometric indices.

## 1 Introduction

Obesity has become a global public health challenge and is strongly associated with premature mortality and a broad range of cardiometabolic disorders, including cardiovascular disease (CVD), type 2 diabetes, non-alcoholic fatty liver disease, and several cancers (1–8). Because excess adiposity and adverse body fat distribution are potentially modifiable risk factors, anthropometric assessment remains an important component of clinical evaluation and population-based risk surveillance (9).

Traditional anthropometric indices, including body mass index (BMI), waist circumference (WC), and waist-to-hip ratio (WHR), are widely used to assess obesity-related health risk. BMI is simple and reproducible, but it does not distinguish between fat mass and lean mass and provides limited information on regional fat distribution (4, 10). WC and WHR better reflect abdominal adiposity and visceral fat accumulation, which are more closely linked to cardiometabolic risk; however, these measures are also correlated with overall body size and may not fully separate the contributions of central adiposity, general adiposity, and reduced muscle mass (11, 12). These limitations have encouraged interest in alternative anthropometric indices that may capture different aspects of body composition.

Weight-adjusted waist index (WWI), calculated as WC divided by the square root of body weight, has been proposed as an anthropometric measure that reduces dependence on body weight while retaining information on central adiposity (13, 14). Previous studies suggest that higher WWI may reflect a phenotype characterized by relatively greater abdominal adiposity and lower lean mass. Accordingly, WWI has been associated with several obesity-related conditions, including CVD, hypertension, psoriasis, and diabetes (15–18). Unlike BMI, which primarily reflects total body mass, WWI may therefore provide complementary information on the balance between central adiposity and body weight. However, whether WWI is consistently associated with hard clinical endpoints such as all-cause and CV mortality across cohort studies remains uncertain.

Several important knowledge gaps remain. First, although individual cohort studies have examined the association between WWI and mortality, their findings vary in magnitude across populations, data sources, and adjustment strategies. Second, the dose–response pattern of the association between WWI and mortality has not been clearly established. In particular, it remains uncertain whether the association is best described as linear or whether there is evidence of nonlinearity across the WWI continuum. Third, although previous systematic reviews and meta-analyses have evaluated traditional or related anthropometric indices such as BMI, WC, WHR, body roundness index, and a body shape index (4, 19), WWI differs from these measures in its mathematical adjustment for body weight and its proposed ability to reflect central adiposity relative to body mass. Therefore, a focused synthesis of cohort evidence specifically evaluating WWI and mortality outcomes is warranted.

To address these gaps, we conducted a systematic review and meta-analysis of cohort studies evaluating the association between WWI and all-cause and CV mortality. The objectives were: (1) to quantify the association between WWI and the risk of all-cause and CV mortality; (2) to assess the linear and nonlinear dose–response relationships between WWI and mortality outcomes; and (3) to explore whether study-level characteristics, including population type, region, follow-up, and adjustment for BMI and other covariates, may contribute to heterogeneity.

## 2 Methods

This systematic review and meta-analysis was conducted in accordance with the 2020 *Preferred Reporting Items for Systematic Reviews and Meta-Analyses* (PRISMA) guidelines (20), see Appendix Table 1. The study used previously published, de-identified data and therefore did not require approval from an institutional review board. And it was registered with PROSPERO.^1^

### 2.1 Literature search strategy

This study conducted a comprehensive search of the PubMed, Embase, Web of Science, Scopus and Medline databases from their establishment until February 2025. In April 2026, the databases were updated for another search, aiming to comprehensively screen cohort studies published in peer-reviewed journals that explored the association between WWI and the risk of all-cause or CV mortality. The core PubMed search strategy was as follows: (“clinical outcomes” OR mortality OR “all-cause mortality” OR “cardiovascular mortality” OR “CV mortality”) AND (“weight-adjusted waist index” OR WWI).

The full search strategies for all databases are provided in Appendix Table 2. Reference lists of relevant studies and reviews were also manually screened to identify additional eligible articles. Two reviewers (YP and HN) independently screened titles, abstracts, and full texts. Disagreements were resolved by discussion or by consultation with a third reviewer (LZ).

### 2.2 Selection criteria

Studies were included if they met the following criteria: (1) Cohort study design; (2) Published in English; (3) WWI identified as the exposure variable; and (4) Reported at least one mortality outcome (all-cause or CV) with corresponding hazard ratios (HRs), relative risks (RRs), and 95% confidence intervals (CIs), or sufficient data to compute these. In addition, animal studies, clinical trials, reviews, letters, and commentaries were excluded.

### 2.3 Handling of overlapping cohorts

To prevent the inflation of participant counts and pseudo-replication, we pre-specified a non-overlapping analytical strategy for our primary analysis. For the primary analysis, each participant population was counted only once. When multiple studies used overlapping waves or participants from the same database, we retained the study that met the following criteria in descending order of priority: the broadest and most representative participant population relevant to the outcome; the largest sample size or number of mortality events; the longest follow-up; the most fully adjusted model, particularly adjustment for age, sex, BMI or other adiposity measures, diabetes, hypertension, CVD, and lifestyle factors; the most recent or methodologically complete publication. Analyses that included all 47 eligible estimates, regardless of population overlap, were strictly designated as secondary/sensitivity analyses.

### 2.4 Data extraction and quality assessment

Two authors (YP and HN) independently extracted data using a standardized form. Extracted information included: first author, year of publication, study location, age, sample size, number of cases, WWI levels and categories, outcome types (all-cause mortality, CV mortality, or both), follow-up, covariates adjusted for, and fully adjusted HRs with 95% CIs. Discrepancies were resolved through discussion with a third author (LZ). Adjusted estimates were prioritized over unadjusted ones. For descriptive purposes, multiple datasets reported within a single study were extracted separately; however, overlapping datasets were not simultaneously included in the primary meta-analysis. If both categorical and continuous WWI estimates were available, we extracted both estimates separately for corresponding analyses. For continuous analyses, estimates were standardized as HRs per cm/ √ kg increment in WWI whenever possible. For categorical analyses, the comparison between the highest and lowest WWI categories was used.

The methodological quality of included cohort studies was assessed independently by two reviewers using the Newcastle-Ottawa Scale (NOS) (21), which evaluates three domains: selection of study groups, comparability of groups, and ascertainment of outcomes. Studies scoring 6.5–9 points were considered high quality, 3.5–6 points moderate quality, and fewer than 3 points low quality. Disagreements were resolved through discussion. The NOS assessment was used to inform the risk-of-bias domain in the Grading of Recommendations Assessment, Development and Evaluation (GRADE) evaluation but was not treated as a direct substitute for the overall certainty of evidence.

### 2.5 Data synthesis and statistical analysis

Pooled HRs with 95% CIs were calculated for all studies. RRs were considered equivalent to HRs, and odds ratios (ORs) were converted to HRs using the Zhang and Yu method (22). Pooled estimates were obtained using an inverse variance–weighted random-effects model. The primary analysis was based on non-overlapping cohorts. Analyses including all eligible study estimates, including potentially overlapping public database-derived reports, were considered secondary and exploratory.

For dose–response analyses, studies were eligible if they reported at least three quantitative WWI categories. Both linear and nonlinear associations were examined using random-effects models. Linear trends in log-HR estimates across WWI categories were analyzed through generalized least squares regression (23), while potential nonlinear dose–response relationships were evaluated using restricted cubic spline functions with three knots (at the 10th, 50th, and 90th percentiles) combined in a multivariate meta-analysis (24). Ninety-five percent confidence intervals were derived from standard errors of linear predictor differences between each WWI level and the reference value, calculated from covariate means and coefficient covariance matrices (25). Nonlinearity was tested using the likelihood ratio test by comparing the nonlinear and linear models (26).

Subgroup analyses were performed according to data source, age, sex, region, and population type with additional adjustment for BMI, CVD, diabetes, hypertension, and physical activity. Random-effects meta-regression was also planned to further evaluate whether study-level characteristics explained between-study heterogeneity. The proportion of between-study variance explained by each moderator was assessed by comparing τ2 before and after inclusion of the moderator. Each subgroup analysis included at least two eligible studies (27). Heterogeneity was assessed using Cochran’s Q test (*p* < 0.10 indicating significant heterogeneity) and quantified with the I^2^ statistic, where 25, 50, and 75% represented low, moderate, and high heterogeneity, respectively (28). Publication bias was examined using Begg’s rank correlation test and Egger’s linear regression test (29, 30), with corrections applied using Duval and Tweedie’s nonparametric trim-and-fill method (31). All statistical analyses were performed using Stata SE version 17.0 (64-bit), with two-tailed *p* values < 0.05 considered statistically significant.

### 2.6 Quality of evidence

We assessed the certainty of evidence for both all-cause and CV mortality outcomes using GRADE framework (32). Because the included studies were observational cohorts, the evidence certainty initially started at “low.” The certainty was then systematically evaluated and potentially downgraded across five domains: Risk of bias: Downgraded if a substantial proportion of information came from studies with high risk of bias (assessed via the NOS); Inconsistency: Downgraded if high, unexplained heterogeneity (I^2^ > 50%) was present and point estimates varied widely across studies; Indirectness: Downgraded if populations, exposures, or outcomes differed significantly from the review’s core objective; Imprecision: Downgraded if the pooled confidence intervals were wide or crossed the threshold of clinical decision-making; Publication bias: Downgraded if funnel plots, statistical tests, or trim-and-fill analyses strongly suggested missing studies that could alter the magnitude or direction of the effect. Evidence could theoretically be upgraded for large magnitude of effect, dose–response gradients, or if residual confounding would have reduced the observed effect.

## 3 Results

### 3.1 Study selection and characteristics

**Figure 1:**
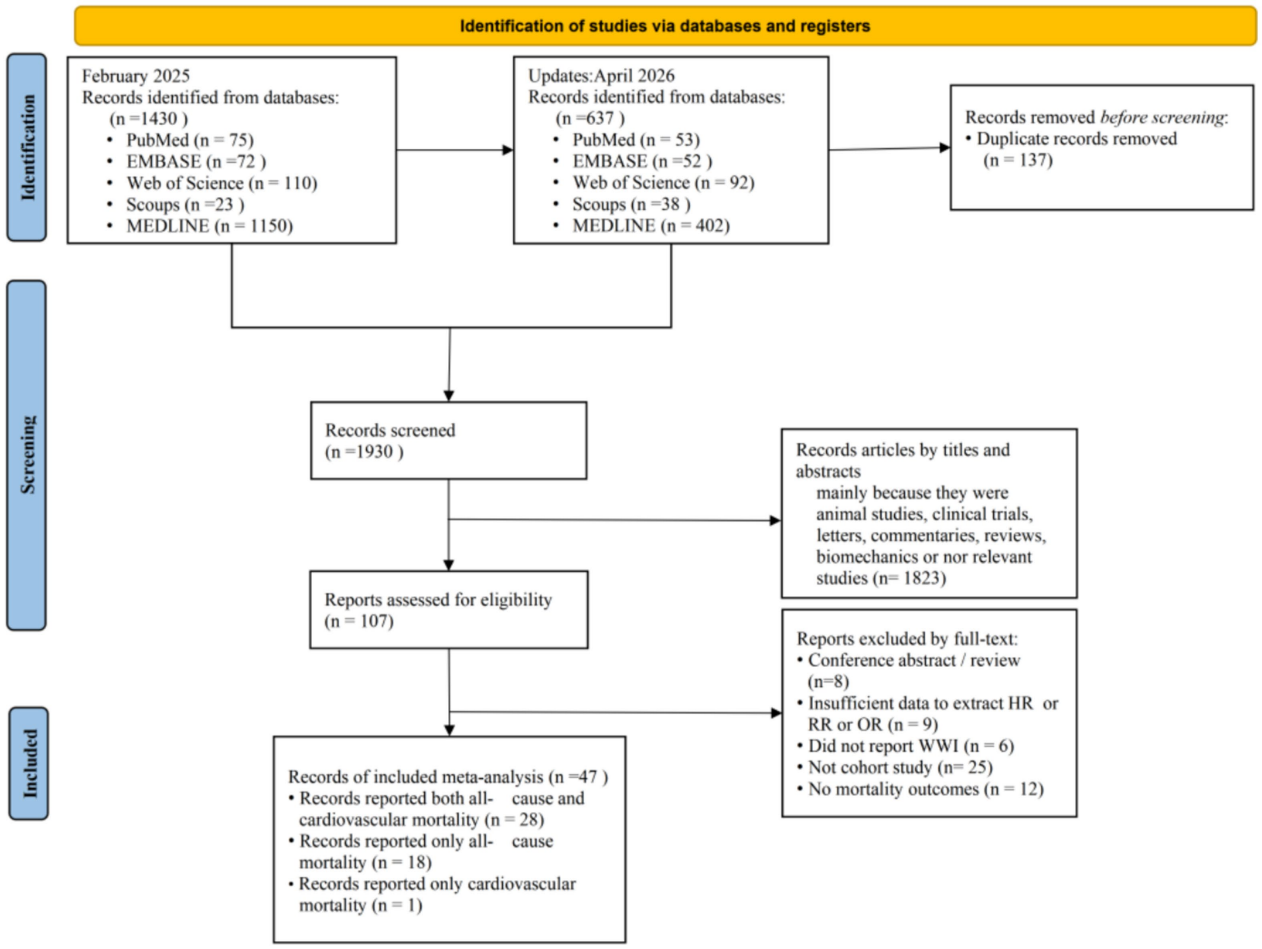
Flow chart of study identification.

A total of 2,067 records were identified from the databases. After removal of duplicates and screening of titles and abstracts, 107 full-text articles were assessed for eligibility. Following detailed evaluation, 47 eligible studies were included in the systematic review. Reasons for full-text exclusion were recorded and are presented in detail in the PRISMA flow diagram (Figure 1).

Several included studies were derived from overlapping public databases, particularly the National Health and Nutrition Examination Survey (NHANES). According to our prespecified analytical approach to avoid pseudo-replication, we retained only the largest and most comprehensive NHANES-based estimate (Peng & Zhang, 2025 (33)) for the primary analysis and combined it with studies from independent, non-overlapping data sources. The full set of 47 eligible studies (50 cohorts) was retained for descriptive synthesis and secondary/exploratory analyses.

Across all eligible reports combined, the summed sample size was 446,218 participants (62,855 all-cause deaths and 11,722 CV deaths). However, because several reports were derived from overlapping public database populations, this figure represents the total sample size across study reports rather than the count of unique individuals. The primary non-overlapping analysis included 9 independent cohorts with 14,133 participants for all-cause mortality and 8 independent cohorts with 4,007 participants for CV mortality, see Appendix Table 3.

Studies were published between 2020 and 2026 and were predominantly conducted in the US. (*n* = 37), China (*n* = 7), the United Kingdom. (*n* = 3), and other regions. Sample sizes ranged from 367 to 86,169 participants. Quality assessment using the NOS yielded scores ranging from 6 to 9 (mean = 8.35), indicating generally good methodological quality (Appendix Table 4).

### 3.2 Association between WWI and mortality

#### 3.2.1 Primary analysis

##### 3.2.1.1 All-cause mortality

**Figure 2:**
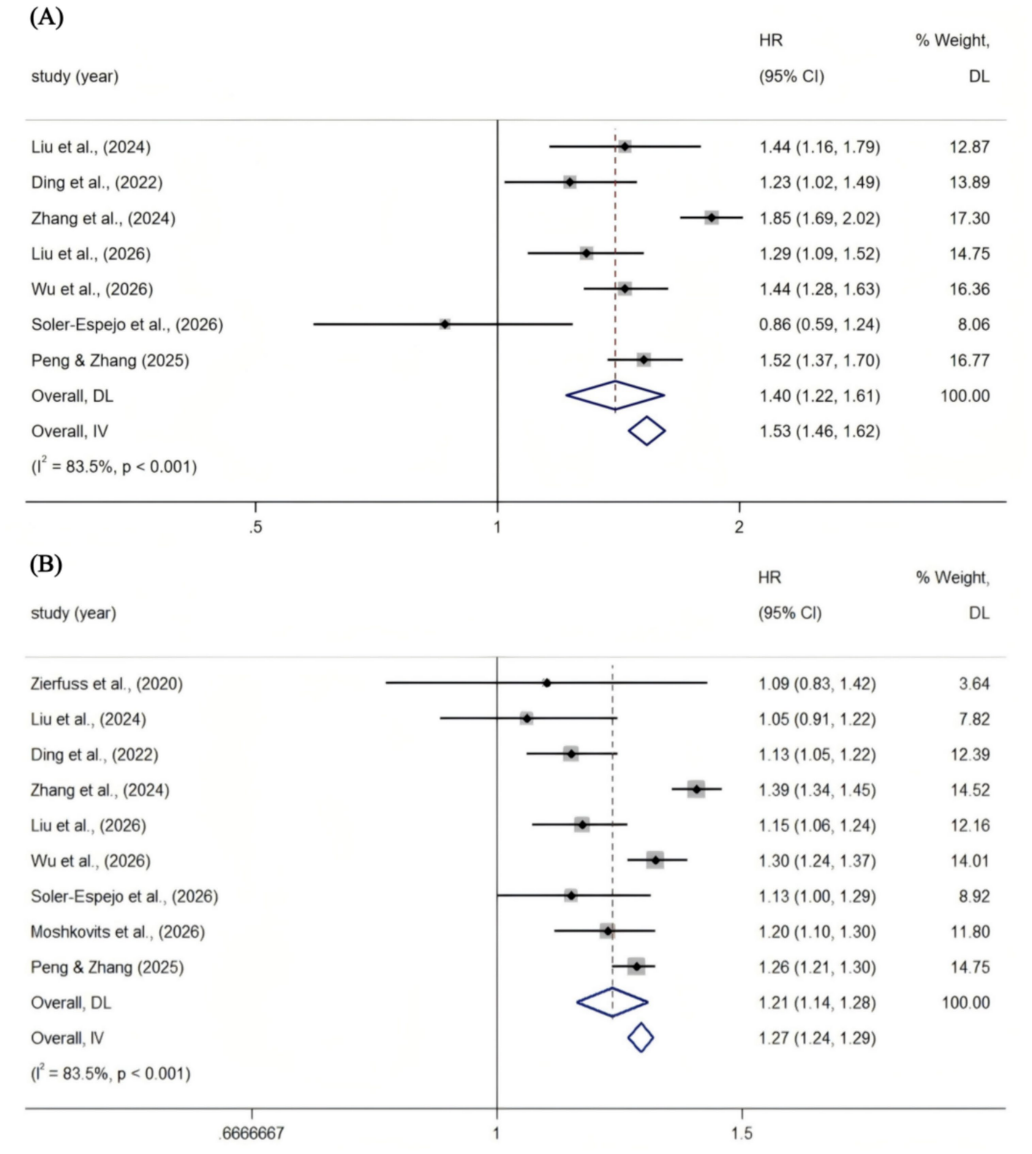
Association between WWI and the risk of all-cause mortality. **(A)**: the highest versus lowest WWI categories, **(B)**: continuous exposure (per 1 cm/ √ kg increment in WWI).

For the highest versus lowest WWI categories, the pooled HR was 1.40 (95% CI: 1.22–1.61, *p* < 0.001) with *I*^2^ = 83.5% (Figure 2A).

For continuous exposure (per 1 cm/ √ kg increment in WWI), each unit increase was associated with a pooled HR of 1.21 (95% CI: 1.14–1.28, *p* < 0.001), corresponding to a 21% increased risk of all-cause mortality. Heterogeneity was *I*^2^ = 83.5% (Figure 2B).

##### 3.2.1.2 CV mortality

**Figure 3:**
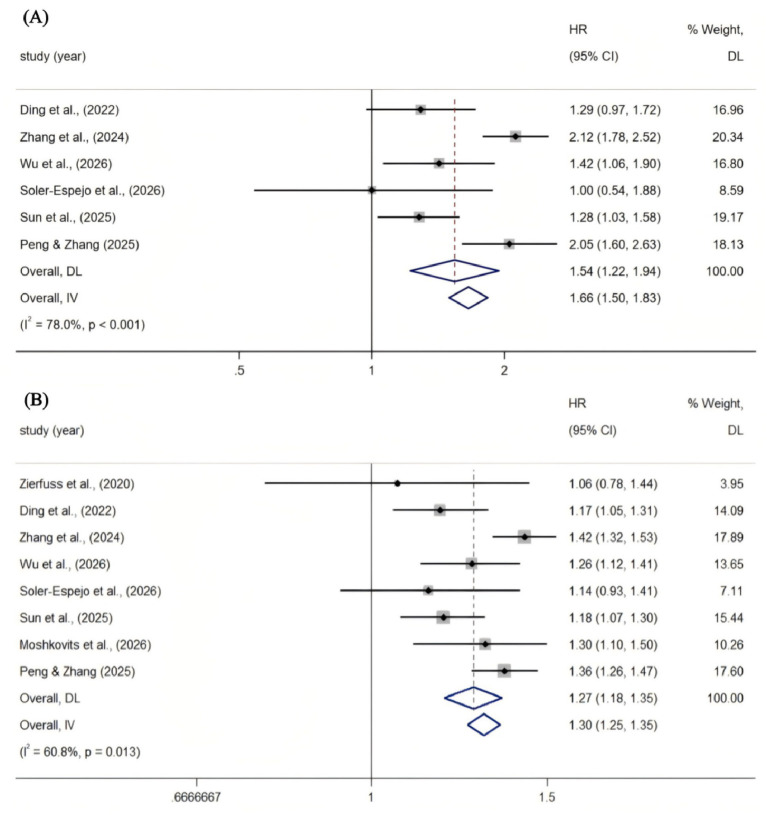
Association between WWI and the risk of CV mortality. **(A)**: the highest versus lowest WWI categories, **(B)**: continuous exposure (per 1 cm/ √ kg increment in WWI).

For the highest versus lowest WWI categories, the pooled HR was 1.54 (95% CI: 1.22–1.94, *p* < 0.001) with substantial heterogeneity (*I*^2^ = 78.0%; Figure 3A).

For continuous exposure (per 1 cm/ √ kg increment in WWI), each unit increase was associated with a pooled HR of 1.27 (95% CI: 1.18–1.35, *p* < 0.001), corresponding to a 27% increased risk of CV mortality. Heterogeneity was I^2^ = 60.8% (Figure 3B).

#### 3.2.2 Secondary analysis

**Figure 4:**
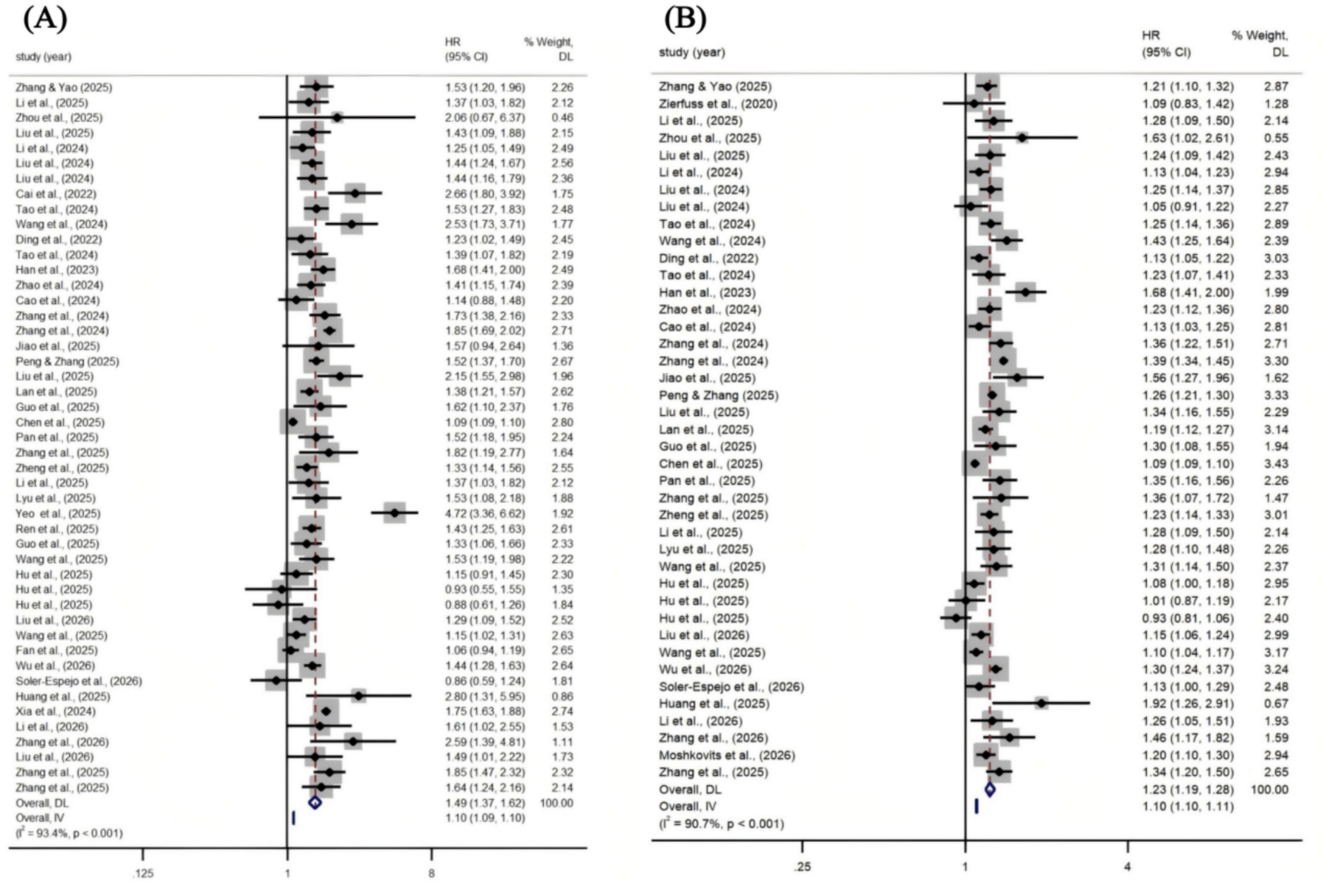
Forest plot of pooled relative risk of all-cause mortality. **(A)**: the highest versus lowest WWI categories, **(B)**: continuous exposure (per 1 cm/ √ kg increment in WWI).

For all-cause mortality (highest vs. lowest category), the pooled HR was 1.49 (95% CI: 1.37–1.62, *p* < 0.001) with high heterogeneity (*I*^2^ = 93.4%; Figure 4A).

For continuous exposure (per 1 cm/ √ kg increment), the pooled HR was 1.23 (95% CI: 1.19–1.28, *p* < 0.001) with high heterogeneity (*I*^2^ = 90.7%; Figure 4B).

**Figure 5:**
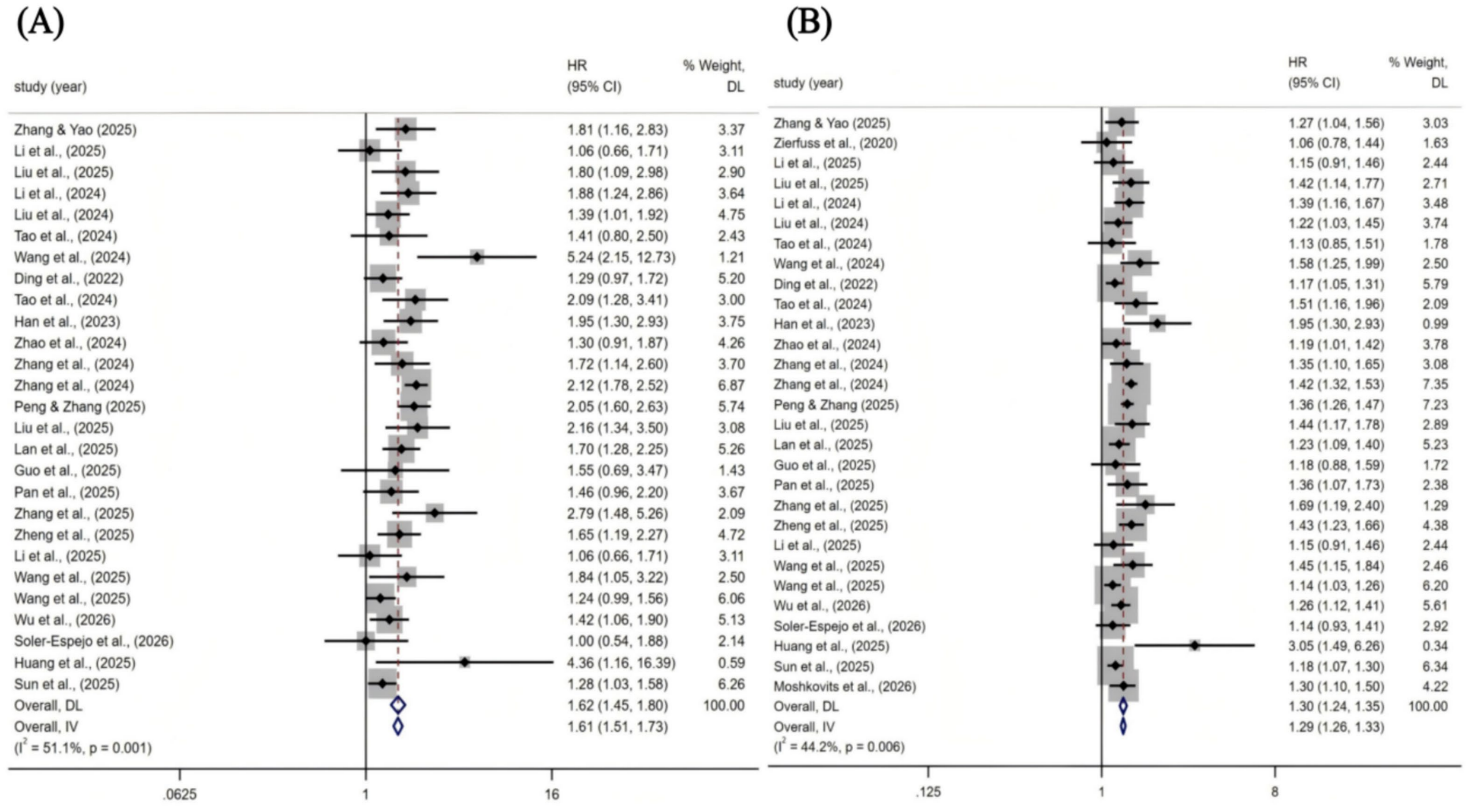
Forest plot of pooled relative risk of CV mortality. **(A)**: the highest versus lowest WWI categories, **(B)**: continuous exposure (per 1 cm/ √ kg increment in WWI).

For CV mortality (highest vs. lowest category), the pooled HR was 1.62 (95% CI: 1.45–1.80, *p* < 0.001) with *I*^2^ = 51.1% (Figure 5A).

For continuous exposure (per 1 cm/ √ kg increment), the pooled HR was 1.29 (95% CI: 1.26–1.33, *p* < 0.001) with *I*^2^ = 44.2% (Figure 5B).

The secondary analysis produced slightly higher effect estimates than the primary non-overlapping analysis, which is consistent with the influence of overlapping public database-derived cohorts on pooled estimates.

### 3.3 Dose–response analysis

**Figure 6:**
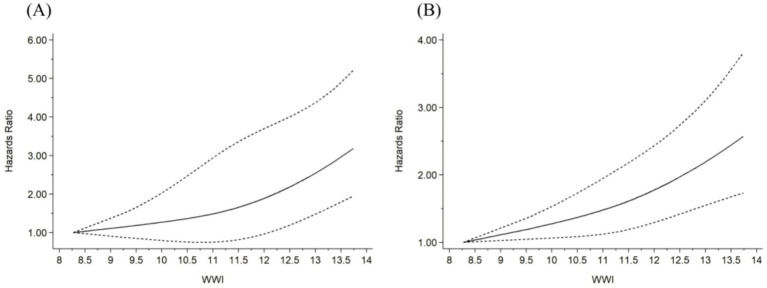
Dose–response relationships for the association between WWI and risk of mortality. **(A)** all-cause mortality, **(B)** CV mortality.

For all-cause mortality dose–response, the formal tests did not provide evidence of nonlinearity for either outcome (*p* = 0.5125); therefore, no statistically supported threshold was identified (Figure 6A; Appendix Table 6). For CV mortality dose–response, the formal test for nonlinearity was also not statistically significant (*p* = 0.4416), the spline curve visually suggests accelerated risk at higher WWI values (Figure 6B; Appendix Table 7).

### 3.4 Subgroup analyses

Subgroup analyses stratified by data source, follow-up, age, sex, region, and population type were conducted, adjusting for BMI, CVD, diabetes, hypertension, and physical activity. No statistically significant subgroup differences were detected, except for the association between WWI and all-cause and CV mortality according to BMI adjustment status(*p* < 0.05) and region (*p* = 0.01). Additional subgroup findings are presented in Table 1.

**Table 1:** Results of subgroup analyses about WWI and the risk of mortality.

Subgroup	Number	HR	95% confidence intervals	*p*	*I*^2^ (%)	*p* value for interaction
All-cause mortality						
Data source						*p* = 0.34
NHANES	33	1.24	1.19–1.29	0.001	87.3%	
Other source	8	1.19	1.11–1.29	< 0.001	85.5%	
Follow-up						*p* = 0.11
<10 years	28	1.21	1.16–1.26	< 0.001	86.1%	
≥10 years	13	1.28	1.21–1.35	< 0.001	79.3%	
Age						*p* = 0.06
<60	20	1.28	1.21–1.36	< 0.001	94.5%	
≥60	21	1.19	1.14–1.25	< 0.001	66.6%	
Sex, proportion						*p* = 0.45
Male, % ≥ 50	17	1.25	1.2–1.31	< 0.001	66.7%	
Male, % < 50	24	1.22	1.16–1.27	< 0.001	89.3%	
Region						*p* = 0.01
The US.	34	1.24	1.19–1.29	< 0.001	87%	
China	2	1.11	1.04–1.19	0.002	0%	
Europe and other	5	1.26	1.16–1.36	< 0.001	79.6%	
Population type						*p* = 0.68
General population	8	1.21	1.13–1.31	< 0.001	83.5%	
CVD	7	1.21	1.16–1.25	< 0.001	0%	
Stroke	2	1.25	1.12–1.39	< 0.001	0%	
Metabolic	3	1.26	1.18–1.35	< 0.001	0%	
Inflammatory	4	1.25	1.09–1.45	0.002	94.8%	
Respiratory	7	1.22	1.05–1.42	0.008	83.5%	
Diabetes	7	1.29	1.21–1.38	< 0.001	73%	
Other chronic diseases	3	1.18	1.11–1.27	< 0.001	39%	
Controlling for BMI						*p* = 0.03
Yes	15	1.3	1.22–1.37	< 0.001	71.9%	
No	26	1.2	1.15–1.25	< 0.001	87.7%	
Controlling for CVD						*p* = 0.33
Yes	19	1.21	1.14–1.28	< 0.001	92.1%	
No	22	1.25	1.21–1.29	< 0.001	44%	
Controlling for hypertension						*p* = 0.78
Yes	28	1.24	1.18–1.31	< 0.001	91.7%	
No	13	1.23	1.2–1.26	< 0.001	37.2%	
Controlling for diabetes						*p* = 0.62
Yes	28	1.24	1.18–1.3	< 0.001	91.6%	
No	13	1.22	1.17–1.27	< 0.001	50.8%	
Controlling for physical activity						*p* = 0.11
Yes	13	1.28	1.21–1.35	< 0.001	76.5%	
No	28	1.21	1.16–1.26	< 0.001	85.6%	
CV mortality						
Data source						*p* = 0.22
NHANES	22	1.31	1.26–1.36	< 0.001	37.5%	
Other source	7	1.24	1.15–1.35	< 0.001	62.1%	
Follow-up						*p* = 0.74
<10 years	19	1.30	1.25–1.35	< 0.001	33.3%	
≥10 years	10	1.28	1.18–1.39	< 0.001	61%	
Age						*p* = 0.46
<60	14	1.3	1.26–1.35	< 0.001	48.9%	
≥60	15	1.27	1.21–1.34	< 0.001	41.9%	
Sex, proportion						*p* = 0.07
Male, % ≥ 50	14	1.36	1.3–1.42	< 0.001	9.3%	
Male, % < 50	15	1.27	1.2–1.35	< 0.001	52.1%	
Region						*p* = 0.01
The US.	22	1.31	1.26–1.36	< 0.001	37.5%	
China	2	1.18	1.09–1.26	< 0.001	0.0%	
Europe and other	5	1.34	1.26–1.41	< 0.001	52.1%	
Population type						*p* = 0.12
General population	6	1.25	1.15–1.36	< 0.001	67.4%	
CVD	8	1.26	1.17–1.34	< 0.001	3.7%	
Metabolic	3	1.34	1.18–1.51	< 0.001	17.6%	
Inflammatory	2	1.3	1.17–1.44	< 0.001	16.4%	
Respiratory	3	1.68	1.4–2.03	< 0.001	31.4%	
Diabetes	5	1.37	1.29–1.45	< 0.001	25.9%	
Other chronic diseases	2	1.33	1.14–1.55	< 0.001	0%	
Controlling for BMI						*p* = 0.01
Yes	10	1.36	1.3–1.43	< 0.001	36.9%	
No	19	1.26	1.22–1.31	< 0.001	39.8%	
Controlling for CVD						*p* = 0.34
Yes	10	1.34	1.23–1.46	< 0.001	62.7%	
No	19	1.28	1.23–1.32	< 0.001	27.4%	
Controlling for hypertension						*p* = 0.70
Yes	19	1.32	1.24–1.4	< 0.001	54.4%	
No	10	1.3	1.23–1.36	< 0.001	15.5%	
Controlling for diabetes						*p* = 0.25
Yes	20	1.33	1.25–1.41	< 0.001	51.2%	
No	9	1.27	1.2–1.33	< 0.001	20.1%	
Controlling for physical activity						*p* = 0.56
Yes	12	1.31	1.23–1.4	< 0.001	52.7%	
No	17	1.28	1.23–1.34	< 0.001	39.6%	

HR, hazard ratio; BMI, body mass index; CV, cardiovascular; NHANES, national health and nutrition examination survey; CVD, cardiovascular diseases.

### 3.5 Meta-regression analyses

Random-effects meta-regression analyses were conducted to explore whether study-level moderators explained between-study heterogeneity. Results are presented in Appendix Table 5. For all-cause mortality, none of the examined moderators significantly explained between-study heterogeneity. For CV mortality, most moderators were also not statistically significant. One exploratory comparison suggested a stronger association in respiratory disease populations than in general population cohorts ( β = 0.334, SE = 0.153, 95% CI: 0.034 to 0.634, *p* = 0.029). However, this finding was based on a limited number of studies and multiple exploratory comparisons. Therefore, it is impossible to fully explain the observed differences and consistent factors.

### 3.6 Sensitivity analyses

Leave-one-out sensitivity analyses were performed by sequentially removing each study and recalculating the pooled estimate. No single study substantially changed the overall associations, indicating that the findings were not driven by any single outlier study. And restriction to studies with lower risk of bias did not materially alter the pooled estimates.

### 3.7 Publication bias

**Figure 7:**
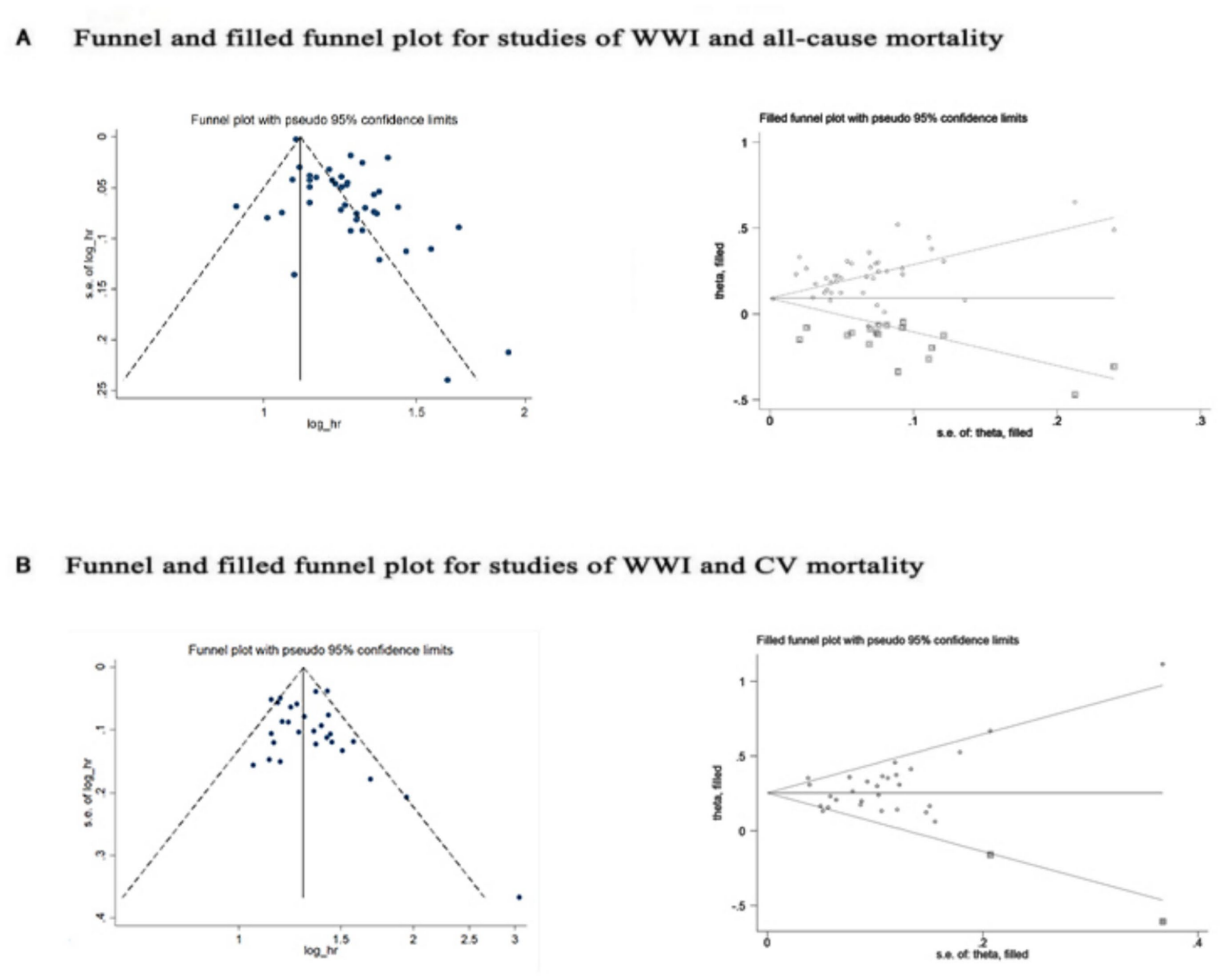
Funnel and filled funnel plot for studies of WWI and all-cause and CV mortality. **(A)** all-cause mortality, **(B)** CV mortality.

Funnel plots for all-cause and CV mortality showed notable asymmetry. For all-cause mortality, Egger’s (*p* = 0.719) and Begg’s (*p* = 0.559) tests indicated no publication bias. The trim-and-fill method confirmed result stability, with an adjusted HR of 1.12 (95% CI, 1.08–1.16; random effects model, *p* < 0.001), and 19 studies imputed (Figure 7A). This marked attenuation suggests that the original estimate for all-cause mortality may have been influenced by small-study effects, selective reporting, or publication bias. The current evidence base may overestimate the true association between WWI and all-cause mortality. For CV mortality, Egger’s (*p* = 0.364) and Begg’s (*p* = 0.165) tests similarly detected no publication bias. The trim-and-fill method identified two potentially missing studies needed for funnel plot symmetry; the adjusted pooled HR remained consistent with the original estimate (Figure 7B), suggesting less evidence of publication bias for CV mortality than for all-cause mortality.

### 3.8 Grading the evidence

As summarized in Table 2, because all included studies were cohort studies, the initial certainty of evidence was rated as low. For all-cause mortality, the certainty of evidence was rated as low because of substantial heterogeneity, potential publication bias. For CV mortality, the certainty of evidence was rated as moderate because the association remained positive in the non-overlapping primary analysis, but certainty was limited by heterogeneity, and observational design.

**Table 2:** GRADE assessments of evidence.

Outcome	No. of studies	Downgrade quality of evidence					Upgrade quality of evidence			Overall quality of evidence
		**Risk of bias**	**Inconsistency**	**Indirectness**	**Imprecision**	**Publication bias**	**Large effect**	**Dose-response**	**Plausible confounding**	
All-cause mortality	38	no serious^a^	serious^b^	no serious^c^	no serious^d^	serious^e^	No^f^	Yes^g^	Yes^h^	ÅÅOO Low
CV mortality	28	no serious^i^	serious^j^	no serious^k^	no serious^l^	no serious^m^	No^n^	Yes^o^	Yes^p^	ÅÅÅO Moderate

Grading of Recommendations Assessment Development and Evaluation (GRADE).

^a^Observational design (initial rating: low).

^b^Substantial heterogeneity (*I*^2^ = 90.7% in secondary analysis, *I*^2^ = 83.5% in primary continuous analysis), downgraded.

^c^In 38 studies (41 cohorts), controlling for BMI was associated with a higher risk of all-cause mortality compared to not controlling for BMI, considering that central fat accumulation has already exerted significant negative effects on the metabolic system. However, all two groups showed a similar positive relationship in our analysis. (HR: 1.3, 95% CI: 1.22–1.37, *n* = 15; HR: 1.2, 95% CI: 1.15–1.25, *n* = 26;). Not downgraded.

^d^Optimal information size met (62,855 all-cause mortality events), with the 95% CI excluding the null value. Not downgraded.

^e^Trim-and-fill analysis imputed 19 studies and attenuated the estimate to HR 1.12, substantially below the original pooled HR of 1.49, downgraded.

^f^Pooled HR < 2. Not upgraded.

^g^The analysis revealed a significant positive linear association between WWI and all-cause mortality. Upgraded.

^h^The risk of all-cause mortality was higher after adjusting for diabetes, with controlling for diabetes (HR: 1.24) surpassing no controlling for diabetes (HR: 1.22), suggesting unmeasured confounding may underestimate the true association and warranting a higher certainty of evidence. Upgraded.

## 4 Discussion

In this systematic review and meta-analysis of cohort studies, we found that higher WWI was positively associated with an increased risk of all-cause and CV mortality. In our primary non-overlapping analysis, each 1 cm/ √ kg increment in WWI was associated with a 21% higher risk of all-cause mortality and a 27% higher risk of CV mortality. Dose–response analyses indicated that WWI serves as an anthropometric marker associated with elevated mortality risk. However, for all-cause mortality, the trim-and-fill analysis imputed 19 potentially missing studies and substantially attenuated the pooled hazard ratio from 1.49 to 1.12. This marked attenuation suggests that small-study effects, selective reporting, or publication bias may have inflated the original estimates (34). Although Egger’s and Begg’s tests were not statistically significant, the presence of substantial funnel plot asymmetry and the substantial trim-and-fill attenuation indicate that the true association may be considerably more modest than suggested by the original pooled estimate. For cardiovascular mortality, evidence of publication bias was less pronounced, with trim-and-fill imputing only two potentially missing studies.

Considerable heterogeneity was observed across studies, particularly for all-cause mortality (I^2^ = 83.5% in the primary non-overlapping analysis). Although subgroup and sensitivity analyses provided some insights, meta-regression did not identify a single study-level factor that fully explained this heterogeneity. This persistent heterogeneity limits the precision and generalizability of the pooled estimates.

Notably, subgroup analyses revealed that region and adjustment for BMI were associated with differences in the magnitude of the WWI and mortality association. The magnitude of the association between WWI and both all-cause and CV mortality was consistently lower in Chinese populations compared with studies conducted in the U. S. and Europe. This difference likely stems from regional variations in obesity phenotypes (35). Western populations generally exhibit a higher prevalence of extreme obesity and severe sarcopenic obesity (36, 37). Consequently, a unit increase in WWI in these populations may capture a more extreme pathophysiological progression of visceral adiposity and metabolic dysfunction than in Asian cohorts, where severe obesity is less common (35, 38). Furthermore, Asian populations are known to develop cardiometabolic complications at lower absolute thresholds of adiposity (39). Studies that adjusted for BMI also showed larger WWI and mortality associations than those without BMI adjustment, suggesting that WWI captures aspects of body composition that are statistically independent of general adiposity.

Because WWI may represent a functional marker of body composition quality rather than an anatomical measurement alone. WWI is calculated as WC divided by the square root of body weight (40). Therefore, a higher WWI may reflect relatively larger abdominal circumference for a given body weight. This phenotype may indicate greater central or visceral adiposity relative to overall mass and may also be consistent with lower lean mass or sarcopenic obesity in some individuals (41–43). Visceral adiposity is metabolically active and has been linked to insulin resistance, chronic low-grade inflammation, oxidative stress, endothelial dysfunction, dyslipidemia, and activation of neurohormonal pathways (44, 45). These processes may contribute to atherosclerosis, cardiac remodeling, vascular injury, and metabolic dysregulation, which in turn increase the risk of mortality (46). In addition, lower muscle mass may worsen glucose metabolism, reduce physical reserve, and increase vulnerability to frailty, disability, and adverse outcomes during chronic illness (47, 48). The construction of WWI may partly capture this imbalance between abdominal adiposity and body weight. Unlike BMI, which increases with both fat mass and lean mass, WWI may be higher when WC is disproportionately large relative to body weight (49, 50). This feature could explain why WWI remains associated with mortality in some studies after adjustment for BMI (51). However, the present meta-analysis did not directly compare WWI with imaging-based measures of visceral fat or lean mass, nor did it evaluate whether WWI outperforms simpler anthropometric measures. Future mechanistic studies in well-characterized populations with detailed body composition assessment are needed.

This study has several strengths. First, it systematically synthesized cohort evidence on the association between WWI and all-cause and CV mortality. Second, both continuous and categorical WWI analyses were evaluated. Third, dose–response analyses were conducted to assess the shape of the association. Fourth, in response to concerns about overlapping public database-derived cohorts, we performed a non-overlapping primary analysis that retained only one estimate from overlapping NHANES-derived studies and combined it with independent non-NHANES cohorts. Fifth, heterogeneity was explored using subgroup analyses, sensitivity analyses, leave-one-out analyses, and random-effects meta-regression.

Several important limitations must be explicitly acknowledged. First, a large proportion of the available evidence was derived from overlapping public databases, particularly NHANES. Although we mitigated the risk of pseudo-replication by prespecifying a primary analysis based solely on non-overlapping cohorts, residual dependence or overlap in the secondary analyses cannot be completely ruled out. Second, there was evidence of substantial publication bias and small-study effects, particularly for all-cause mortality. The trim-and-fill analysis imputed 19 potentially missing studies, leading to a marked attenuation of the pooled HR from 1.49 to 1.12. This dramatic attenuation suggests that the original pooled estimate likely overestimated the true magnitude of the association. Third, and crucially, our study synthesized association estimates (HRs) and did not evaluate predictive performance metrics such as discrimination (e.g., C-statistic) or reclassification (e.g., Net Reclassification Improvement or Integrated Discrimination Improvement). Consequently, while WWI is a marker associated with mortality, the current evidence does not demonstrate whether adding WWI to traditional models improves individual risk prediction or clinical decision-making.

Future research should focus on several key areas. First, individual participant data analyses are needed to examine whether WWI has consistent associations across age, sex, ethnicity, BMI category, and clinical subgroups. Second, prediction-model studies are needed to determine whether WWI improves discrimination, calibration, reclassification, and clinical decision-making beyond established risk factors. Third, future studies should avoid duplicate use of overlapping public databases or clearly specify how overlapping waves and participant samples are handled. Finally, potential WWI thresholds should only be proposed after statistically supported nonlinear analyses and external validation.

## 5 Conclusion

In conclusion, this systematic review and meta-analysis indicates that a higher WWI is associated with an increased risk of all-cause and CV mortality. Based on current observational evidence, WWI should be considered an anthropometric marker associated with mortality risk rather than a validated predictive tool. Furthermore, the dose–response relationship is compatible with a linear pattern, and present data do not provide statistical support for a definitive risk threshold or cut-off. Further independent, prospective studies with formal evaluations of predictive performance, such as discrimination, calibration, and reclassification analyses, are required to determine whether the integration of WWI improves clinical risk stratification beyond established anthropometric measures like BMI and WC.

## Data Availability

The original contributions presented in the study are included in the article/Supplementary material, further inquiries can be directed to the corresponding author.

## Footnotes

1 CRD420251001504; available at https://www.crd.york.ac.uk/PROSPERO/view/CRD420251001504
