# Correction: Dose–response analysis between weight-adjusted waist index and all-cause and cardiovascular mortality: a systematic review and meta-analysis

**DOI:** 10.3389/fnut.2026.1918461

**Published:** 2026-08-03

**Authors:** Pengyu Yang, Neng Huang, Jing Ye, Mingxin He, Yao Wu, Dan Liu, Hua Zhang, Ping Lei Chui, Zuoyan Liu

**Affiliations:** 1 Department of Rehabilitation Medicine, West China School of Nursing, Sichuan University, Chengdu, China; 2 Department of Critical Care Medicine, Peking University Shenzhen Hospital, Shenzhen, China; 3 Department of Nursing, Leshan Vocational and Technical College, Leshan, China; 4 Department of Psychiatry, Ziyang Psychosis Hospital, Ziyang, China; 5 School of Nursing, Hainan Medical University, Haikou, China; 6 Department of Nursing Science, Faculty of Medicine, Universiti Malaya, Kuala Lumpur, Malaysia

**Keywords:** all-cause mortality, cardiovascular mortality, dose–response, meta-analysis, weight adjusted waist index, WWI

In the published article, Table 2 was mistakenly included in the Supplementary material. Table 2 has now been added to the main article. The in-text citation for “Appendix Table 2” in section **3 Results**, subsection *3.8 Grading the evidence*, paragraph 1 has been revised to “Table 2.”

The original version of this article has been updated.

**Table 2:** GRADE assessments of evidence.

Outcome	No. of studies	Downgrade quality of evidence					Upgrade quality of evidence			Overall quality of evidence
		Risk of bias	Inconsistency	Indirectness	Imprecision	Publication bias	Large effect	Dose-response	Plausible confounding	
All-cause mortality	38	No serious^a^	Serious^b^	No serious^c^	No serious^d^	Serious^e^	No^f^	Yes^g^	Yes^h^	ÅÅOO low
CV mortality	28	No serious^i^	Serious^j^	No serious^k^	No serious^l^	No serious^m^	No^n^	Yes^o^	Yes^p^	ÅÅÅO moderate

Grading of Recommendations Assessment Development and Evaluation (GRADE).

^*a*^Observational design (initial rating: low).

^*b*^Substantial heterogeneity (I^2^ = 90.7% in secondary analysis, I^2^ = 83.5% in primary continuous analysis), downgraded.

^*c*^In 38 studies (41 cohorts), controlling for BMI was associated with a higher risk of all-cause mortality compared to not controlling for BMI, considering that central fat accumulation has already exerted significant negative effects on the metabolic system. However, all two groups showed a similar positive relationship in our analysis. (HR: 1.3, 95% CI: 1.22–1.37, n = 15; HR: 1.2, 95% CI: 1.15–1.25, n = 26;). Not downgraded.

^*d*^Optimal information size met (62,855 all-cause mortality events), with the 95% CI excluding the null value. Not downgraded.

^*e*^Trim-and-fill analysis imputed 19 studies and attenuated the estimate to HR 1.12, substantially below the original pooled HR of 1.49, downgraded.

^*f*^Pooled HR < 2. Not upgraded.

^*g*^The analysis revealed a significant positive linear association between WWI and all-cause mortality. Upgraded.

^*h*^The risk of all-cause mortality was higher after adjusting for diabetes, with controlling for diabetes (HR: 1.24) surpassing no controlling for diabetes (HR: 1.22), suggesting unmeasured confounding may underestimate the true association and warranting a higher certainty of evidence. Upgraded.

^*i*^Observational design (initial rating: low).

^*j*^Moderate heterogeneity (I^2^ = 60.8% in primary continuous analysis, I^2^ = 44.2% in secondary analysis), downgraded.

^*k*^In 28 studies (29 cohorts), controlling for BMI was associated with a higher risk of CV mortality compared to not controlling for BMI, considering that central fat accumulation has already exerted significant negative effects on the metabolic system. However, all two groups showed a similar positive relationship in our analysis. (HR: 1.36, 95% CI: 1.3–1.43, n = 10; HR: 1.26, 95% CI: 1.22–1.31, n = 19). Not downgraded.

^*l*^Optimal information size met (11,722 CV mortality events), with the 95% CI excluding the null value. Not downgraded.

^*m*^Although the trim-and-fill method identified two potentially missing studies needed for funnel plot symmetry, the adjusted pooled HR remained consistent with the original estimate; Egger's (P = 0.364) and Begg's (P = 0.165) tests similarly detected no publication bias. Not downgraded.

^*n*^Pooled HR < 2. Not upgraded.

^*o*^The analysis revealed a significant positive linear association between WWI and CV mortality. Upgraded.

^*p*^The risk of CV mortality was greater in controlling for physical activity (HR: 1.31) compared to not controlling for physical activity (HR: 1.28), suggesting unmeasured confounding may underestimate the true association and warranting a higher certainty of evidence. Upgraded.

