# Supplementary material for: Dose–response analysis between weight-adjusted waist index and all-cause and cardiovascular mortality: a systematic review and meta-analysis
Source: Front Nutr. 2026 Jun 24;13:1766501. doi: 10.3389/fnut.2026.1766501 (PMC13341855; doi:10.3389/fnut.2026.1766501)
Supplement: Supplementary file 1 [file Data_Sheet_1.docx]

Contents

[Appendix Table 1 PRISMA CHECKLIST 2](#_Toc22175)

[Appendix Table 2 Search strategies and results of this study. 5](#_Toc4858)

[Appendix Table 3 Characteristics of 50 cohorts on WWI and risk of all-cause and CV mortality. 6](#_Toc8323)

[Appendix Table 4 Quality scores of included studies according to Newcastle-Ottawa Scale for cohort studies. 19](#_Toc24206)

[Appendix Table 5 Random-effects meta-regression analyses 21](#_Toc9516)

[Appendix Table 6 Predicted Hazard Ratios and 95% Confidence Intervals for All-Cause Mortality 24](#_Toc28931)

[Appendix Table 7 Predicted Hazard Ratios and 95% Confidence Intervals for CV Mortality 24](#_Toc2202)

[References 25](#_Toc493)

# **Appendix Table 1** PRISMA CHECKLIST

| **Section and Topic** | **Item #** | **Checklist item** | **Location where item is reported** |
| --- | --- | --- | --- |
| **TITLE** | | |  |
| Title | 1 | Identify the report as a systematic review. | Page 1 |
| **ABSTRACT** | | |  |
| Abstract | 2 | See the PRISMA 2020 for Abstracts checklist. | Pages 1 |
| **INTRODUCTION** | | |  |
| Rationale | 3 | Describe the rationale for the review in the context of existing knowledge. | Page 2 |
| Objectives | 4 | Provide an explicit statement of the objective(s) or question(s) the review addresses. | Page 2-3 |
| **METHODS** | | |  |
| Eligibility criteria | 5 | Specify the inclusion and exclusion criteria for the review and how studies were grouped for the syntheses. | Pages 3 |
| Information sources | 6 | Specify all databases, registers, websites, organisations, reference lists and other sources searched or consulted to identify studies. Specify the date when each source was last searched or consulted. | Pages 3 |
| Search strategy | 7 | Present the full search strategies for all databases, registers and websites, including any filters and limits used. | Pages 3; Appendix Table 2 |
| Selection process | 8 | Specify the methods used to decide whether a study met the inclusion criteria of the review, including how many reviewers screened each record and each report retrieved, whether they worked independently, and if applicable, details of automation tools used in the process. | Page 3 |
| Data collection process | 9 | Specify the methods used to collect data from reports, including how many reviewers collected data from each report, whether they worked independently, any processes for obtaining or confirming data from study investigators, and if applicable, details of automation tools used in the process. | Page 3 |
| Data items | 10a | List and define all outcomes for which data were sought. Specify whether all results that were compatible with each outcome domain in each study were sought (e.g. for all measures, time points, analyses), and if not, the methods used to decide which results to collect. | Page 3-4 |
|  | 10b | List and define all other variables for which data were sought (e.g. participant and intervention characteristics, funding sources). Describe any assumptions made about any missing or unclear information. | Page 3-4 |
| Study risk of bias assessment | 11 | Specify the methods used to assess risk of bias in the included studies, including details of the tool(s) used, how many reviewers assessed each study and whether they worked independently, and if applicable, details of automation tools used in the process. | Pages 4 |
| Effect measures | 12 | Specify for each outcome the effect measure(s) (e.g. risk ratio, mean difference) used in the synthesis or presentation of results. | Pages 4 |
| Synthesis methods | 13a | Describe the processes used to decide which studies were eligible for each synthesis (e.g. tabulating the study intervention characteristics and comparing against the planned groups for each synthesis (item #5)). | Pages 4 |
|  | 13b | Describe any methods required to prepare the data for presentation or synthesis, such as handling of missing summary statistics, or data conversions. | Pages 4 |
|  | 13c | Describe any methods used to tabulate or visually display results of individual studies and syntheses. | Pages 4 |
|  | 13d | Describe any methods used to synthesize results and provide a rationale for the choice(s). If meta-analysis was performed, describe the model(s), method(s) to identify the presence and extent of statistical heterogeneity, and software package(s) used. | Pages 4 |
|  | 13e | Describe any methods used to explore possible causes of heterogeneity among study results (e.g. subgroup analysis, meta-regression). | Pages 4 |
|  | 13f | Describe any sensitivity analyses conducted to assess robustness of the synthesized results. | Pages 4 |
| Reporting bias assessment | 14 | Describe any methods used to assess risk of bias due to missing results in a synthesis (arising from reporting biases). | Pages 3-4 |
| Certainty assessment | 15 | Describe any methods used to assess certainty (or confidence) in the body of evidence for an outcome. | Pages 4-5 |
| **RESULTS** | | |  |
| Study selection | 16a | Describe the results of the search and selection process, from the number of records identified in the search to the number of studies included in the review, ideally using a flow diagram. | Page 5; Figure [1](https://pmc.ncbi.nlm.nih.gov/articles/PMC7174309/#Fig1) |
|  | 16b | Cite studies that might appear to meet the inclusion criteria, but which were excluded, and explain why they were excluded. | Pages 5; Figure [1](https://pmc.ncbi.nlm.nih.gov/articles/PMC7174309/#Fig1) |
| Study characteristics | 17 | Cite each included study and present its characteristics. | Pages 5 |
| Risk of bias in studies | 18 | Present assessments of risk of bias for each included study. | Pages 6; Appendix Table 4 |
| Results of individual studies | 19 | For all outcomes, present, for each study: (a) summary statistics for each group (where appropriate) and (b) an effect estimate and its precision (e.g. confidence/credible interval), ideally using structured tables or plots. | Pages 6-13 |
| Results of syntheses | 20a | For each synthesis, briefly summarise the characteristics and risk of bias among contributing studies. | Pages 13 |
|  | 20b | Present results of all statistical syntheses conducted. If meta-analysis was done, present for each the summary estimate and its precision (e.g. confidence/credible interval) and measures of statistical heterogeneity. If comparing groups, describe the direction of the effect. | Pages 6-12 |
|  | 20c | Present results of all investigations of possible causes of heterogeneity among study results. | Pages 10-12 |
|  | 20d | Present results of all sensitivity analyses conducted to assess the robustness of the synthesized results. | Pages 6-7; 12 |
| Reporting biases | 21 | Present assessments of risk of bias due to missing results (arising from reporting biases) for each synthesis assessed. | Pages 13 |
| Certainty of evidence | 22 | Present assessments of certainty (or confidence) in the body of evidence for each outcome assessed. | Pages 14; Table 2 |
| **DISCUSSION** | | |  |
| Discussion | 23a | Provide a general interpretation of the results in the context of other evidence. | Pages 14 |
|  | 23b | Discuss any limitations of the evidence included in the review. | Pages 15 |
|  | 23c | Discuss any limitations of the review processes used. | Pages 14-16 |
|  | 23d | Discuss implications of the results for practice, policy, and future research. | Pages 15 |
| **OTHER INFORMATION** | | |  |
| Registration and protocol | 24a | Provide registration information for the review, including register name and registration number, or state that the review was not registered. | Page 3 |
|  | 24b | Indicate where the review protocol can be accessed, or state that a protocol was not prepared. | Page 3 |
|  | 24c | Describe and explain any amendments to information provided at registration or in the protocol. | Page 3 |
| Support | 25 | Describe sources of financial or non-financial support for the review, and the role of the funders or sponsors in the review. | Pages 16 |
| Competing interests | 26 | Declare any competing interests of review authors. | Page 16 |
| Availability of data, code and other materials | 27 | Report which of the following are publicly available and where they can be found: template data collection forms; data extracted from included studies; data used for all analyses; analytic code; any other materials used in the review. | Page 16 |

*From:*  Page MJ, McKenzie JE, Bossuyt PM, Boutron I, Hoffmann TC, Mulrow CD, et al. The PRISMA 2020 statement: an updated guideline for reporting systematic reviews. BMJ 2021;372:n71. doi: 10.1136/bmj.n71

For more information, visit: <http://www.prisma-statement.org/>

# Appendix Table 2 Search strategies and results of this study.

1. PubMed

| **Search number** | **Search terms** | **Search results and time** |
| --- | --- | --- |
| 1 | (clinical outcomes OR mortality OR all-cause mortality OR cardiovascular mortality OR CV mortality ) AND (Weight-adjusted waist index OR WWI) | 128 |

(b) Embase

| **Search number** | **Search terms** | **Search results** |
| --- | --- | --- |
| 1 | ('clinical outcomes':ab,ti OR 'mortality':ab,ti OR 'all-cause mortality':ab,ti OR 'CV mortality':ab,ti OR 'cardiovascular mortality':ab,ti) AND ('weight-adjusted waist index':ab,ti OR 'WWI':ab,ti) | 124 |

(c) WOS

| **Search number** | **Search terms** | **Search results** |
| --- | --- | --- |
| 1 | (clinical outcomes OR mortality OR all-cause mortality OR cardiovascular mortality OR CV mortality ) AND (Weight-adjusted waist index OR WWI) | 202 |

(d) Scopus

| **Search number** | **Search terms** | **Search results** |
| --- | --- | --- |
| 1 | (clinical outcomes OR mortality OR all-cause mortality OR cardiovascular mortality OR CV mortality ) AND (Weight-adjusted waist index OR WWI) | 61 |

(e) Ovid MEDLINE

| **Search number** | **Search terms** | **Search results** |
| --- | --- | --- |
| 1 | (clinical outcomes OR mortality OR all-cause mortality OR cardiovascular mortality OR CV mortality ) AND (Weight-adjusted waist index OR WWI) | 1552 |

# Appendix Table 3 **Characteristics of 50 cohorts on WWI and risk of all-cause and CV mortality.**

|  | **Study year** | **Location** | **Population** | **Sex/Age** | **Number of Cases**  **(A/C/N)** | **WWI**  **cm/√kg** | **Sample Size**  **(A/C/N)** | **Endpoints**  **(mortality)** | **Data Source** | **Follow-up**  **(Years)** | **Adjustment** |
| --- | --- | --- | --- | --- | --- | --- | --- | --- | --- | --- | --- |
| 1 | Zhang & Yao, 2025^[1]^ | the US. | Hyperlipidemia | Male: 47.4%  49.9 ± 16.0 | 1454/380/12, 785 | NA | NA | All-cause | NHANES 2003–2018 | 8.4 | Age, sex, race, education level, marital status, PIR, alcohol drink, smoking, physical activity, hypertension, diabetes, heart disease, and CKD |
|  |  |  |  |  |  |  |  | CV |  |  |  |
| 2 | Zierfuss et al., 2020^[2]^ | Austria | Peripheral artery disease | Male: 66.5%  73 ± 9 | 57/36/367 | NA | NA | All-cause | Austrian Medical University of Vienna2006-2013 | 5.06 | Age, serum creatinine, CRP, diabetes status, gender, LDL-C, [systolic blood pressure](https://www.sciencedirect.com/topics/medicine-and-dentistry/systolic-blood-pressure), smoking status and history of peripheral revascularisation |
|  |  |  |  |  |  |  |  | CV |  |  |  |
| 3 | Li et al., 2025^[3]^ | the US. | CVD | Male: 53%  64.18 ± 14.13 | 2024/674/4, 445 | < 11.03 | 346/116/1009 | All-cause | NHANES 1999–2018 | 6.8 | Age, gender, race, education, family income-poverty ratio, smoking and drinking history, BMI, weight, WC, SBP, DBP, urine creatinine, FBG, HbA1c, TC, TG, HDL-C, LDL-C, platelet count, neutrophils count and lymphocyte count |
|  |  |  |  |  |  | 11.03-11.53 | 480/171/1090 |  |  |  |  |
|  |  |  |  |  |  | 11.53-12.05 | 543/181/1150 | CV |  |  |  |
|  |  |  |  |  |  | > 12.05 | 655/206/1196 |  |  |  |  |
| 4 | Zhou et al., 2025^[4]^ | the US. | Psoriasis | Male: 48.46%  45.58 ±17.74 | 1678/NA/19, 919 | NA | NA | All-cause | NHANES 2003–2006 & 2009–2014 | 8.4 | Age, gender, race, education level, marital status, PIR, drinking, smoking, moderate activity, diabetes, hypertension, CVD, LDL-C and total cholesterol. |
| 5 | Liu et al., 2025^[5]^ | the US. | Stroke | Male: 53%  65.057±0.475 | 624/251/  1, 427 | <11.021 | NA/NA/357 | All-cause | NHANES 1999–2018 | 6.9 | Age, sex, race, education, PIR and marital status, smoking, alcohol consumption, physical activity, diabetes mellitus, hypertension and CHD |
|  |  |  |  |  |  | 11.021-11.538 | NA/NA/357 |  |  |  |  |
|  |  |  |  |  |  | 11.538-12.047 | NA/NA/356 | CV |  |  |  |
|  |  |  |  |  |  | >12.047 | NA/NA/357 |  |  |  |  |
| 6 | Li et al., 2024^[6]^ | the US. | Cancer | Male: 47%  57.59 ± 15.79 | 1547/322/4, 463 | <10.85 | 283/NA/1116 | All-cause | NHANES 1999–2018 | 7.3 | Age, sex, race, BMI, education, smoking, activity, diabetes, high blood pressure, coronary heart disease, PIR, HDL/LDL |
|  |  |  |  |  |  | 10.85-11.3 | 389/NA/1115 |  |  |  |  |
|  |  |  |  |  |  | 11.38-11.89 | 438/NA/1116 | CV |  |  |  |
|  |  |  |  |  |  | >11.89 | 437/NA/1116 |  |  |  |  |
| 7 | Liu et al., 2024^[7]^ | the US. | Metabolic dysfunction-related fatty liver disease | Male: 53%  49.757 ± 0.221 | 2462/818/15, 694 | <11.1 | NA/NA/5232 | All-cause | NHANES 1999–2018 | 9 | Age, sex, race/ethnicity, education, PIR, and marital status, smoking, physical activity, dietary energy intake, diabetes mellitus, hypertension, CVD, CKD, TG, TC, and HDL-C, BMI |
|  |  |  |  |  |  | 11.1-11.8 | NA/NA/5232 |  |  |  |  |
|  |  |  |  |  |  | ≥11.8 | NA/NA/5230 | CV |  |  |  |
| 8 | Liu et al., 2024^[8]^ | China | General population | Male: 42%  54.3 ± 14.8 | 1554/NA/21, 750 | NA | 154 /NA/1554 | All-cause | Zhejiang metabolic  syndrome cohort 2010-2014 | 9.31 | Age, sex, smoking status, drinking status, and baseline BMI |
|  |  |  |  |  |  |  | 260/NA/1554 |  |  |  |  |
|  |  |  |  |  |  |  | 391/NA/1554 |  |  |  |  |
|  |  |  |  |  |  |  | 749/NA/1554 |  |  |  |  |
| 9 | Cai et al., 2022^[9]^ | China | General population | Male: 41%  71.10±9.32 | 339/NA/  1, 863 | <10.68 | 66/NA/621 | All-cause | Wanshou road community of haidian district  in Beijing 2009-2010 | 10.8 | Age, sex, BMI, WC, SBP, DBP, FPG, TC, HDL, LDL, TG, SUA, SCr, smoking, alcohol drinking, CHD, HTN, diabetes, stroke |
|  |  |  |  |  |  | 10.68-11.24 | 108/NA/617 |  |  |  |  |
|  |  |  |  |  |  | ≥11.25 | 165/NA/625 |  |  |  |  |
| 10 | Tao et al., 2024^[10]^ | the US. | Diabetes | Male: 51%  58.74 | 2130/596/8, 005 | 8.85-11.12 | 407/111/2002 | All-cause | NHANES 1999–2020 | 11 | Age, gender and race, marriage, education, smoking status, alcohol user, SBP, DBP, eGFR, ALT, AST, fast glucose, HbA1c, TC, TG, HDL-C, LDL-C |
|  |  |  |  |  |  | 11.12-11.61 | 508/137/1999 |  |  |  |  |
|  |  |  |  |  |  | 11.61-12.09 | 569/175/2001 | CV |  |  |  |
|  |  |  |  |  |  | 12.09-15.39 | 646/173/2003 |  |  |  |  |
| 11 | Wang et al., 2024^[11]^ | the US. | Asthma | Male: 36%  46.42±0.42 | 565/164/  3, 223 | 8.51-10.57 | NA/NA/806 | All-cause | NHANES 1999–2018 | 8.6 | Age, gender, race, education level, marital status, and PIR, smoking, alcohol consumption, and physical activity, diabetes, hypertension, CVD, and family history of asthma |
|  |  |  |  |  |  | 10.57-11.20 | NA/NA/806 |  |  |  |  |
|  |  |  |  |  |  | 11.20-11.82 | NA/NA/805 | CV |  |  |  |
|  |  |  |  |  |  | >11.82 | NA/NA/806 |  |  |  |  |
| 12 | Ding et al., 2022^[12]^ | China | General population | Male: 41%  59.0 ± 13.3 | 838/390/  12, 447 | <10.1 | 191/83/3112 | All-cause | Urban and rural areas of Jiangxi Province 2013-2014 | 5.6 | Age, sex, area, physical activity, smoking status, alcohol drinking status, hypertension, stroke, and antihypertensive drugs |
|  |  |  |  |  |  | 10.1-10.7 | 179/78/3111 |  |  |  |  |
|  |  |  |  |  |  | 10.7-11.2 | 178/83/3111 | CV |  |  |  |
|  |  |  |  |  |  | ≥11.2 | 290/146/3113 |  |  |  |  |
| 13 | Tao et al., 2024^[13]^ | the US. | Metabolic syndrome | Male: 51%  52.23 | 2464/677/12, 641 | 9.22-11.06 | 352/94/3158 | All-cause | NHANES 1999–2020 | 8.3 | Age, gender and race, marriage, education, smoking status, alcohol user, IPR, SBP, DBP, eGFR, ALT, AST, FBG, HbA1c, TC, TG, HDL-C, LDL-C, diabetes, hypertension, CKD |
|  |  |  |  |  |  | 11.06-11.53 | 525/139/3157 |  |  |  |  |
|  |  |  |  |  |  | 11.53-12.00 | 714/206/3169 | CV |  |  |  |
|  |  |  |  |  |  | 12.00-14.79 | 873/238/3157 |  |  |  |  |
| 14 | Han et al., 2023^[14]^ | the US. | General population | Male: 49%  46.38±15.79 | 1870/349/26, 882 | NA | NA | All-cause | NHANES 2005–2014 | 5.8 | Gender, age, race, education level, urinary albumin, urinary creatinine, ALT, AST, total cholesterol, serum creatinine, triglycerides, serum uric acid, BMI, SBP, DBP, fasting plasma glucose, HDL-C, LDL-C, smoking, hypertension and diabetes status |
|  |  |  |  |  |  |  |  | CV |  |  |  |
| 15 | Zhao et al., 2024^[15]^ | the US. | Diabetes | Male: 47%  57.0±14.68 | 1083/360/6, 651 | NA | 174/57/1638 | All-cause | NHANES 2005–2018 | 13 | Gender, race, age, drinking status, smoking status, education levels, with or without hypertension, BMI, and PIR |
|  |  |  |  |  |  |  | 253/89/1637 |  |  |  |  |
|  |  |  |  |  |  |  | 300/100/1638 | CV |  |  |  |
|  |  |  |  |  |  |  | 356/114/1638 |  |  |  |  |
| 16 | Cao et al., 2024^[16]^ | the US. | General population | Male: 49%  47.99±18.52 | 1065/NA/18, 592 | 9.56-10.34 | 94/NA/4648 | All-cause | NHANES 2011-2018 | 4.8 | Age, gender, race, education level, ratio of family income to poverty, alcohol drinking status, smoking status, stroke status, coronary heart disease status, renal failure status, liver diseases status, myocardial infarction status, and cancer status |
|  |  |  |  |  |  | 10.58-10.92 | 191/NA/4648 |  |  |  |  |
|  |  |  |  |  |  | 11.16-11.5 | 303/NA/4648 |  |  |  |  |
|  |  |  |  |  |  | 11.73-12.55 | 477/NA/4648 |  |  |  |  |
| 17 | Zhang et al., 2024^[17]^ | the US. | Diabetes | Male: 52%  59.7 | 1722/503/6, 825 | NA | NA | All-cause mortality | NHANES 1999-2018 | 8.2 | Age, sex, race, education, ratio of family income to poverty, alcohol intake, smoking status and physical activity, duration of diabetes, duration of diabetes, use of diabetes medication, use of hypertensive drugs, eGFR, body mass index, total cholesterol, high-density lipoprotein, HbA1c, any hypertension and any CVD |
|  |  |  |  |  |  |  |  | CV mortality |  |  |  |
|  |  | The UK. | Diabetes | Male: 59%  59.1 | 5796/1743/31, 615 | NA | NA | All-cause | UK Biobank 2006-2010 | 14.8 | Age, sex, race, Townsend deprivation index, alcohol intake, smoking status, and physical activity, duration of diabetes, duration of diabetes, use of diabetes medication, use of hypertensive drugs, eGFR, body mass index, total cholesterol, high-density  lipoprotein, HbA1c, any hypertension and any CVD |
|  |  |  |  |  |  |  |  | CV |  |  |  |
| 18 | Jiao et al., 2025^[18]^ | the US. | General population | Male: 47%  44.4±15.5 | 543/NA/9841 | ≤ 10.35 | NA | All-cause | NHANES 2007–2012 | 8–10 | Age, sex, race, education attainment, marital status, family PIR, BMI, smoking status, and physical activity |
|  |  |  |  |  |  | 10.35 – 10.90 |  |  |  |  |  |
|  |  |  |  |  |  | 10.90 – 11.45 |  |  |  |  |  |
|  |  |  |  |  |  | ≥ 11.45 |  |  |  |  |  |
| 19 | Peng & Zhang, 2025^[19]^ | the US. | General population | Male: 48%  49.24 ± 16.90 | NA/NA/45130 | 8.11–10.44 | NA/NA/11284 | All-cause | NHANES 2005–2020 | 7.5 | Age, gender, educational level, marital status, family poverty income, smoking and drinking rates |
|  |  |  |  |  |  | 10.45–11.03 | NA/NA/11284 |  |  |  |  |
|  |  |  |  |  |  | 11.04–11.62 | NA/NA/11281 | CV |  |  |  |
|  |  |  |  |  |  | 11.63–15.70 | NA/NA/11281 |  |  |  |  |
| 20 | Liu et al., 2025^[20]^ | the US. | Rheumatoid arthritis | Male: 42%  58 | 738/242/2564 | 8.37–10.88 | 126/41/640 | All-cause | NHANES 1999–2018 | 8.5 | Age, gender, race, marital status, education, PIR, smoking, drinking, CHD, diabetes, hypertension, blood lipids, liver enzymes, blood sugar, HbA1c |
|  |  |  |  |  |  | 10.88–11.38 | 166/54/643 |  |  |  |  |
|  |  |  |  |  |  | 11.38–11.94 | 209/63/640 | CV |  |  |  |
|  |  |  |  |  |  | 11.94–14.21 | 237/84/641 |  |  |  |  |
| 21 | Lan et al., 2025^[21]^ | the US. | Hypertension | Male: 49%  57 ± 15 | 3190/1052/14350 | 9.91-10.69 | 484/140/3588 | All-cause | NHANES 2003–2016 | 9 ± 4.08 | Sex and age, race, education level, PIR,  drinking and smoking status, diabetes, cardiovascular disease, TC and HDL-C. |
|  |  |  |  |  |  | 10.95-11.25 | 681/242/3586 |  |  |  |  |
|  |  |  |  |  |  | 11.46-11.74 | 905/304/3588 | CV |  |  |  |
|  |  |  |  |  |  | 11.95-12.75 | 1120/366/3588 |  |  |  |  |
| 22 | Guo et al., 2025^[22]^ | the US. | Osteoarthritis | Male: 36%  61 | 611/159/3554 | NA | NA | All-cause | NHANES 1999–2018 | 8-10 | Age, sex, education level, marital status, PIR, race, obesity, smoking, drinking, hypertension, diabetes, and Hyperlipidemia |
|  |  |  |  |  |  |  |  | CV |  |  |  |
| 23 | Chen et al., 2025^[23]^ | the US. | Psoriasis | Male: 47%  48.84 ± 16.38 | 69/NA/577 | 8.82–10.54 | 6/NA/144 | All-cause | NHANES 1999–2018 | 9.4 | Gender, age, race, survey cycle, education level, marital status,  hypertension, total cholesterol, triglycerides, diabetes, smoking history, as well as histories of  arthritis and CVD |
|  |  |  |  |  |  | 10.54–11.10 | 9/NA/144 |  |  |  |  |
|  |  |  |  |  |  | 11.10–11.66 | 19/NA/144 |  |  |  |  |
|  |  |  |  |  |  | 11.66–13.32 | 35/NA/145 |  |  |  |  |
| 24 | Pan et al., 2025^[24]^ | the US. | Diabetes | Male: 53%  62.07 ± 12.45 | 446/163/1247 | 9.15–11.33 | 114/42/416 | All-cause | NHANES 1999–2018 | 6.75 | Gender, age, race, PIR, smoking history, education level, alcohol use, hypertension, hyperlipidemia, CKD, duration of diabetes |
|  |  |  |  |  |  | 11.34–11.96 | 158/54/415 |  |  |  |  |
|  |  |  |  |  |  | 11.97–14.20 | 174/67/416 | CV |  |  |  |
| 25 | Zhang et al., 2025^[25]^ | the US. | COPD | Male: 52%  63 | 371/109/1396 | 9.09-11.03 | 83/16/465 | All-cause | NHANES 1999–2018 | 10 | Age, gender, race, education level, and marital status, BMI; PIR, smoking, drinking, creatinine, uric acid , total energy intake, diabetes, hypertension, cardiovascular disease and cancer |
|  |  |  |  |  |  | 11.03-11.78 | 116/38/465 | CV |  |  |  |
|  |  |  |  |  |  | 11.78-13.85 | 172/55/466 |  |  |  |  |
| 26 | Zheng et al., 2025^[26]^ | the US. | Hypertension | Male: 55%  57.1 ± 15.6 | 2144/615/11556 | <10.96 | 470/127/3852 | All-cause | NHANES 2001–2018 | 8.3 | Age, gender, race/ethnicity, education, BMI, smoking status, drinking status, diabetes, CHD, angina, MI, stroke, CHF, uric acid, albumin, AST, ALT, HDL-C, TC, SBP, DBP. |
|  |  |  |  |  |  | 10.96 – 11.63 | 680/205/3852 | CV |  |  |  |
|  |  |  |  |  |  | > 11.63 | 994/283/3852 |  |  |  |  |
| 27 | Li et al., 2025^[27]^ | the US. | CVD | Male: 53%  64.18 ± 14.13 | 2024/674/4445 | < 11.03 | 346/116/1009 | All-cause | NHANES 1999–2018 | 6.75 | Age, gender, race, education, family income-poverty ratio, smoking and drinking history, BMI, weight, WC, SBP, DBP, urine creatinine, FBG, HbA1c, TC, TG, HDL-C, LDL-C, platelet count, neutrophils count and lymphocyte count |
|  |  |  |  |  |  | 11.03–11.53 | 480/171/1090 |  |  |  |  |
|  |  |  |  |  |  | 11.53 – 12.05 | 543/181/1150 |  |  |  |  |
|  |  |  |  |  |  | > 12.05 | 655/206/1196 | CV |  |  |  |
| 28 | Lyu et al., 2025^[28]^ | the US. | Osteoporosis | Male: 22%  68.19 ± 9.77 | 400/NA/1324 | 10.1-10.9 | 99/NA/331 | All-cause | NHANES 2005–2018 | 10.8 | BMI, gender, age, race, serum calcium, serum phosphorus, serum creatinine, BUN, LDL, history of diabetes, history of hypertension, history of smoking, history of alcohol, education, PIR, and history of bone fracture |
|  |  |  |  |  |  | 11.1-11.5 | 90/NA/331 |  |  |  |  |
|  |  |  |  |  |  | 11.6-12 | 103/NA/331 |  |  |  |  |
|  |  |  |  |  |  | 12.2-13 | 108/NA/331 |  |  |  |  |
| 29 | Yeo et al., 2025^[29]^ | the US. | Metabolic associated fatty liver disease | Male: 48%  42 | 730/NA/2150 | < 10.40 | NA | All-cause | NHANES III 1988–1994, 2017–2020, Kailuan Cohort 2006–2021, UK Biobank Cohort 2014–2017 | 26 | Sex, race/ethnicity, hypertension, DM/preDM, drinks per day, height, weight, hip and FIB-4 score |
|  |  |  |  |  |  | 10.40–10.98 |  |  |  |  |  |
|  |  |  |  |  |  | 10.98–11.51 |  |  |  |  |  |
|  |  |  |  |  |  | > 11.51 |  |  |  |  |  |
| 30 | Ren et al., 2025^[30]^ | China | General population | Male: 46%  84.35 ± 10.88 | 2020/NA/7034 | < 10.58 | 479/NA/1768 | All-cause | CLHLS 2011–2018 | 7 | Age, gender, ethnic, birth place, education, marital, econnomic state, medical insurance, waist, sleep quality, health |
|  |  |  |  |  |  | 10.58 ~ 11.38 | 478/NA/1765 |  |  |  |  |
|  |  |  |  |  |  | 11.38 ~ 12.30 | 461/NA/1749 |  |  |  |  |
|  |  |  |  |  |  | ≥ 12.30 | 602/NA/1752 |  |  |  |  |
| 31 | Guo et al., 2025^[31]^ | the US. | Heart failure | Male: 54%  65.53 ±13.39 | 700/NA/1366 | NA | NA | All-cause | NHANES 1999–2018 | 5.8 | Age, gender, race, marital, education, drink, smoke, hemoglobin A1c, high-density lipoprotein cholesterol, glucose, total cholesterol, triglyceride. |
| 32 | Wang et al., 2025^[32]^ | the US. | Cardiometabolic syndrome | Male: 50%  53.66±15.41 | 1027/292/6506 | NA | 132/39/1627 | All-cause | NHANES 2003–2018 | 7.6 | Age, gender, race, education level, PIR, marital status, alcohol consumption, smoking status, eGFR, history of cancer |
|  |  |  |  |  |  |  | 207/59/1626 |  |  |  |  |
|  |  |  |  |  |  |  | 304/82/1626 | CV |  |  |  |
|  |  |  |  |  |  |  | 384/112/1627 |  |  |  |  |
| 33 | Hu et al., 2025^[33]^ | the US. | Obstructive sleep apnoea | Male: 54%  84.45 (2.03) | 920/NA/7702 | NA | NA | All-cause | NHANES 2005-2008, 2015-2020 | 7 | Sex, age, race, education level, family poverty-income ratio, diabetes, hypertension, cardiovascular disease, chronic kidney disease, smoking status, and alcohol consumption |
|  |  | the US. | Asthma | Male: 43%  71.62 (4.29) | 267/NA/2231 | NA | NA | All-cause | NHANES 2005-2008, 2015-2020 | 6 | Sex, age, race, education level, family poverty-income ratio, diabetes, hypertension, cardiovascular disease, chronic kidney disease, smoking status, and alcohol consumption |
|  |  | the US. | COPD | Male: 43%  71.82 (3.34) | 314/NA/1083 | NA | NA | All-cause | NHANES 2005-2008, 2015-2020 | 6 | Sex, age, race, education level, family poverty-income ratio, diabetes, hypertension, cardiovascular disease, chronic kidney disease, smoking status, and alcohol consumption |
| 34 | Liu et al., 2026^[34]^ | the US. & Canada | Diabetes | Male: 62%  62.72 ± 6.54 | 1274/NA/8182 | 8.403-10.560 | 259/NA/2042 | All-cause | Action to Control Cardiovascular Risk in Diabetes 2001-2009, 2011-2014 | 6.61 | Sex, race, age, education, living alone, history of cardiovascular disease, previous hypertension, proteinuria, heart failure, depression, smoking and alcohol consumption, duration of diabetes, SBP, DBP, FPG, HbA1c, TG, LDL-C, HDL-C and eGFR, the medications use, diuretics, ARBs/ACEIs, CCBs, beta-blockers, biguanides, thiazolidinediones, insulins and aspirin |
|  |  |  |  |  |  | 10.56- 11.045 | 279/NA/2049 |  |  |  |  |
|  |  |  |  |  |  | 11.045-11.570 | 345/NA/2045 |  |  |  |  |
|  |  |  |  |  |  | 11.570-15.093 | 391/NA/2046 |  |  |  |  |
| 35 | Wang et al., 2025^[35]^ | the US. | Postmenopausal women | Male: 0%  62.22 ± 0.17 | 2522/783/10001 | 7.90 – 10.78 | NA | All-cause | NHANES 1999–2018 | 10 | Age, ethnicity, PIR, marital status, education level,  smoking history, alcohol use, PA, CVD history, hypertension history, diabetes history, and cancer history |
|  |  |  |  |  |  | 10.78 – 11.34 |  |  |  |  |  |
|  |  |  |  |  |  | 11.34 – 11.89 |  | CV |  |  |  |
|  |  |  |  |  |  | 11.89 – 15.52 |  |  |  |  |  |
| 36 | Fan et al., 2025^[36]^ | China | General population | Male: 80%  83.0 ± 10.0 | NA/NA/2626 | 0–10.42 | NA/NA/662 | All-cause | CLHLS 2011–2018 | 7 | Age, sex, ethnic, marital, place of birth, living pattern, education, wealth, insurance, smoke, drink. |
|  |  |  |  |  |  | 10.42–11.13 | NA/NA/651 |  |  |  |  |
|  |  |  |  |  |  | 11.13–11.88 | NA/NA/658 |  |  |  |  |
|  |  |  |  |  |  | 11.88–24.29 | NA/NA/655 |  |  |  |  |
| 37 | Wu et al., 2026^[37]^ | The UK. | Chronic kidney disease | Male: 49%  61 | 3874/786/22523 | 6.59 – 9.96 | 534/77/5593 | All-cause | UK Biobank 2006–2010 | 12.6 | Age, sex, annual household income, smoking status, alcohol intake, education level, townsend deprivation index, healthy diet score, physical activity, hyperuricemia, hypertension, diabetes mellitus, HDLC, LDLC, Triglycerides, WBC, eGFR, and UACR |
|  |  |  |  |  |  | 9.96 – 10.50 | 780/140/5640 |  |  |  |  |
|  |  |  |  |  |  | 10.50 – 11.10 | 1007/206/5656 |  |  |  |  |
|  |  |  |  |  |  | 11.10 – 14.91 | 1553/363/5634 | CV |  |  |  |
| 38 | Soler-Espejo et al., 2026^[38]^ | Spain | AF | Male: 43%  77 | 274/102/2070 | NA | NA | All-cause | Murcia AF Project III prospective cohort 2016-2023 | 3 | Age, AF type, CHA₂DS₂-VASc score |
|  |  |  |  |  |  |  |  | CV |  |  |  |
| 39 | Huang et al., 2025^[39]^ | the US. | Preserved ratio impaired spirometry | Male: 46%    47.0 ± 0.6 | 166/48/1454 | 10.199 | 33/6/485 | All-cause | NHANES 2007–2012 | 10 | Age, sex, race, PIR, marital statues and educational level  smoke, drinking, Diabetes, Hypertension, psychical activity and CVD |
|  |  |  |  |  |  | 11.192 | 50/15/484 | CV |  |  |  |
|  |  |  |  |  |  | 12.105 | 83/27/485 |  |  |  |  |
| 40 | Xia et al., 2024^[40]^ | China | General population | Male: 79%  58.1 | 10009/NA/86169 | < 9.80 | 1122/NA/21522 | All-cause | Kailuan prospective cohort 2006-2020 | 13.3 | Age, sex, monthly income, education level, marital status, physical activity, current smoking, current drinking, and family history of cardiometabolic diseases |
|  |  |  |  |  |  | 9.80 – 10.32 | 1424/NA/21553 |  |  |  |  |
|  |  |  |  |  |  | 10.32 – 10.87 | 1873/NA/21478 |  |  |  |  |
|  |  |  |  |  |  | ≥ 10.87 | 3011/NA/21616 |  |  |  |  |
| 41 | Sun et al., 2025^[41]^ | China | General population | Male: 46%  47.13 ± 14.27 | NA/771/36039 | ≤ 9.81 | NA/124/9035 | CV | China Chronic Disease and Risk Factor Surveillance | 5.91 | Age, gender, diabetes, hypertension, dyslipidemia, excessive red meat intake, insufficient fruit and vegetable intake, prolonged sedentary time, insufficient physical activity level, insufficient sleep time, current smoking and current drinking |
|  |  |  |  |  |  | 9.82 – 10.31 | NA/141/8980 |  |  |  |  |
|  |  |  |  |  |  | 10.32 – 10.84 | NA/183/9017 |  |  |  |  |
|  |  |  |  |  |  | ≥ 10.85 | NA/323/9007 |  |  |  |  |
| 42 | Li et al., 2026^[42]^ | the US. | Stroke | Male: 49%  66 | 360/NA/1134 | 8.75 – 11.06 | 66/NA/NA | All-cause | NHANES 2005–2018 | 5.8 | Age, sex, race, education level, marital status, smoking, alcohol use, diabetes, hypertension, and hypercholesterolemia |
|  |  |  |  |  |  | 11.07 – 11.56 | 93/NA/NA |  |  |  |  |
|  |  |  |  |  |  | 11.56 – 12.08 | 89/NA/NA |  |  |  |  |
|  |  |  |  |  |  | 12.08 – 13.80 | 112/NA/NA |  |  |  |  |
| 43 | Zhang et al., 2026^[43]^ | the US. | Asthma | Male: 35%  47.13 ± 14.27 | 188/NA/1260 | 8.66 – 10.87 | 29/NA/420 | All-cause | NHANES 1999–2018 | 7.9 | Age; gender, race, education level and marital status; BMI; PIR, smoking, drinking, creatinine, total energy intake, diabetes, hypertension, cardiovascular disease and blood relatives had asthma |
|  |  |  |  |  |  | 10.87 – 11.67 | 56/NA/420 |  |  |  |  |
|  |  |  |  |  |  | 11.67 – 13.91 | 103/NA/420 |  |  |  |  |
| 44 | Liu et al., 2026^[44]^ | the US. | Hypercholesterolemia | Male: 45%  53.2 ± 15.0 | 215/NA/1188 | NA | 47/NA/396 | All-cause | NHANES 1999–2018 | 8.4 | Age, gender, race, education, current smoker, current drinker, hypertension,  and diabetes. |
|  |  |  |  |  |  |  | 74/NA/397 |  |  |  |  |
|  |  |  |  |  |  |  | 94/NA/395 |  |  |  |  |
| 45 | Moshkovits et al., 2026^[45]^ | Israel | General population | Male: 51%  72 ± 7 | 466/179/939 | NA | NA | All-cause | Glucose Intolerance, Obesity, and Hypertension study | 13 | Age, sex, origin, smoking status, systolic blood pressure, current physical activity, statin use, low GFR, total cholesterol,  glycemic state, history of myocardial infarction |
|  |  |  |  |  |  |  |  | CV |  |  |  |
| 46 | Zhang et al., 2025^[46]^ | the US. | Diabetes | Male: 54%  62.51 ± 12.54 | NA/NA/1487 | NA | NA | All-cause | NHANES 1999–2004 | 12.3 | Age, gender, race, education level, marital status, poverty status, smoking status, drinking status, cardiovascular disease, hypertension, hypercholesterolemia, HbA1c, UACR, CRP, WBC |
| 47 | Zhang et al., 2025^[47]^ | the UK. | Inflammatory bowel disease | Male: 48%  57.0 ± 8.0 | 591/NA/5107 | 9.33 | 77/NA/1278 | All-cause | UK Biobank 2006–2010 | 14.57 | Age, sex, Townsend deprivation index, education level, ethnicity, smoking status, alcohol drinking, IPAQ, duration of IBD and treatment of IBD |
|  |  |  |  |  |  | 10.06 | 136/NA/1276 |  |  |  |  |
|  |  |  |  |  |  | 10.57 | 158/NA/1276 |  |  |  |  |
|  |  |  |  |  |  | 11.27 | 220/NA/1277 |  |  |  |  |

Noted: A, all-cause mortality; C, cardiovascular (CV) mortality; N, total sample size; PIR, the ratio of income to poverty; LDL-C, low-density lipoprotein; CHD, coronary heart disease; HR, hazard ratio; BMI, body mass index; HDL/LDL, high-density lipoprotein cholesterol/low-density lipoprotein cholesterol; CI, corresponding 95% confidence interval; SBP, systolic blood pressure; DBP, diastolic blood pressure; eGFR, glomerular filtration rate; HbA1c, glycated hemoglobin; ALT, alanine aminotransferase; AST, aspartate aminotransferase; HDL-C, high-density lipoprotein-cholesterol; LDL-C, low-density lipoprotein-cholesterol; CVD, cardiovascular diseases; TC, total cholesterol; CKD, chronic kidney disease; CHF, congestive heart failure; WC, waist circumference; FBG, fasting blood glucose; TG, triglycerides; BUN, blood urea nitrogen; DM, diabetes mellitus; FPG, fasting plasma glucose; ARB, angiotensin receptor blocker; ACEI, angiotensin converting enzyme inhibitors; CCB, calcium channel blockers; PA, physical activity; WBC, white blood cell count; UACR, urine albumin-to-creatinine ratio; AF, atrial fibrillation; GFR, glomerular filtration rate; CRP, ‌c-reactive protein; IPAQ, international physical activity questionnaire; IBD, inflammatory bowel disease; COPD, chronic obstructive pulmonary disease.

# Appendix Table 4 **Quality scores of included studies according to Newcastle-Ottawa Scale for cohort studies.**

| **No.** | **Author (Year)** | **(1)** | **(2)** | **(3)** | **(4)** | **(5)** | **(6)** | **(7)** | **(8)** | **Total Score** |
| --- | --- | --- | --- | --- | --- | --- | --- | --- | --- | --- |
| 1 | Zhang & Yao, 2025 | 1 | 1 | 1 | 0.5 | 1.5 | 1 | 1 | 1 | 8 |
| 2 | Zierfuss et al., 2020 | 1 | 1 | 1 | 1 | 2 | 1 | 1 | 1 | 9 |
| 3 | Li et al., 2025 | 1 | 1 | 1 | 1 | 2 | 1 | 1 | 0 | 8 |
| 4 | Zhou et al., 2025 | 1 | 1 | 0 | 1 | 2 | 1 | 1 | 0 | 7 |
| 5 | Liu et al., 2025 | 1 | 1 | 1 | 1 | 2 | 1 | 1 | 0 | 8 |
| 6 | Li et al., 2024 | 1 | 1 | 1 | 1 | 2 | 1 | 1 | 0 | 8 |
| 7 | Liu et al., 2024 | 1 | 1 | 1 | 1 | 2 | 1 | 1 | 0 | 8 |
| 8 | Liu et al., 2024 | 1 | 1 | 1 | 1 | 2 | 1 | 1 | 0 | 8 |
| 9 | Cai et al., 2022 | 1 | 1 | 1 | 1 | 2 | 1 | 1 | 1 | 9 |
| 10 | Tao et al., 2024a | 1 | 1 | 1 | 0 | 2 | 1 | 1 | 0 | 7 |
| 11 | Wang et al., 2024 | 1 | 1 | 1 | 1 | 2 | 1 | 1 | 1 | 9 |
| 12 | Ding et al., 2022 | 1 | 1 | 1 | 0 | 1 | 0 | 1 | 1 | 6 |
| 13 | Tao et al., 2024b | 1 | 1 | 1 | 0 | 2 | 1 | 1 | 1 | 8 |
| 14 | Han et al., 2023 | 1 | 1 | 1 | 1 | 2 | 1 | 1 | 1 | 9 |
| 15 | Zhao et al., 2024 | 1 | 1 | 1 | 1 | 1 | 1 | 1 | 1 | 8 |
| 16 | Cao et al., 2024 | 1 | 0.5 | 1 | 1 | 1.5 | 1 | 1 | 0.5 | 7.5 |
| 17 | Zhang et al., 2024 | 1 | 0.5 | 1 | 1 | 1 | 1 | 1 | 0 | 6.5 |
| 18 | Jiao et al., 2025 | 1 | 1 | 1 | 0 | 2 | 1 | 1 | 1 | 8 |
| 19 | Peng & Zhang, 2025 | 1 | 1 | 1 | 1 | 2 | 1 | 1 | 1 | 9 |
| 20 | Liu et al., 2025 | 1 | 1 | 1 | 1 | 2 | 1 | 1 | 1 | 9 |
| 21 | Lan et al., 2025 | 1 | 0 | 1 | 0 | 2 | 1 | 1 | 0 | 6 |
| 22 | Guo et al., 2025 | 1 | 1 | 1 | 0 | 2 | 1 | 1 | 0 | 7 |
| 23 | Chen et al., 2025 | 1 | 0 | 1 | 0 | 2 | 1 | 1 | 0 | 6 |
| 24 | Pan et al., 2025 | 1 | 0 | 1 | 1 | 2 | 1 | 1 | 0 | 7 |
| 25 | Zhang et al., 2025 | 1 | 1 | 1 | 1 | 2 | 1 | 1 | 1 | 9 |
| 26 | Zheng et al., 2025 | 1 | 1 | 1 | 0 | 2 | 1 | 1 | 1 | 8 |
| 27 | Li et al., 2025 | 1 | 1 | 1 | 0 | 2 | 1 | 1 | 1 | 8 |
| 28 | Lyu et al., 2025 | 1 | 1 | 1 | 0 | 2 | 1 | 1 | 1 | 8 |
| 29 | Yeo et al., 2025 | 1 | 1 | 1 | 0 | 2 | 1 | 1 | 1 | 8 |
| 30 | Ren et al., 2025 | 1 | 1 | 1 | 1 | 2 | 1 | 1 | 1 | 9 |
| 31 | Guo et al., 2025 | 1 | 1 | 1 | 0 | 2 | 1 | 1 | 1 | 8 |
| 32 | Wang et al., 2025 | 1 | 1 | 1 | 1 | 2 | 1 | 1 | 1 | 9 |
| 33 | Hu et al., 2025 | 1 | 1 | 1 | 1 | 2 | 1 | 1 | 1 | 9 |
| 34 | Liu et al., 2026 | 1 | 1 | 1 | 1 | 2 | 1 | 1 | 1 | 9 |
| 35 | Wang et al., 2025 | 1 | 1 | 1 | 1 | 2 | 1 | 1 | 1 | 9 |
| 36 | Fan et al., 2025 | 1 | 1 | 1 | 1 | 2 | 1 | 1 | 0.5 | 8.5 |
| 37 | Wu et al., 2026 | 1 | 1 | 1 | 1 | 2 | 1 | 1 | 1 | 9 |
| 38 | Soler-Espejo et al., 2026 | 1 | 1 | 1 | 1 | 1 | 1 | 1 | 1 | 8 |
| 39 | Huang et al., 2025 | 1 | 1 | 1 | 1 | 2 | 1 | 1 | 0 | 8 |
| 40 | Xia et al., 2024 | 1 | 1 | 1 | 1 | 2 | 1 | 1 | 0 | 8 |
| 41 | Sun et al., 2025 | 1 | 1 | 1 | 1 | 2 | 1 | 1 | 1 | 9 |
| 42 | Li et al., 2026 | 1 | 1 | 1 | 1 | 2 | 1 | 1 | 0 | 8 |
| 43 | Zhang et al., 2026 | 1 | 1 | 1 | 1 | 1 | 1 | 1 | 0 | 7 |
| 44 | Liu et al., 2026 | 1 | 1 | 1 | 1 | 2 | 1 | 1 | 1 | 9 |
| 45 | Moshkovits et al., 2026 | 1 | 1 | 1 | 1 | 2 | 0 | 1 | 0 | 7 |
| 46 | Zhang et al., 2025 | 1 | 1 | 1 | 1 | 1 | 1 | 1 | 0 | 7 |
| 47 | Zhang et al., 2025 | 1 | 1 | 1 | 1 | 2 | 0 | 0 | 1 | 7 |

Notes: Description of NOS points: (1) Representativeness of the exposed cohort. (2) Selection of the non-exposed cohort. (3) Ascertainment of exposure. (4) Demonstration that outcome of interest is not present at start of study. (5) Comparability of cohorts on the basis of the design or analysis. (6) Assessment of outcome. (7) Duration of follow-up period. (8) Adequacy of follow-up.

# Appendix Table 5 **Random-effects meta-regression analyses.**

| **Outcome** | **Moderator** | **Comparison** | **Coefficient** $\text{β}$ | **SE** | **95% CI** | **P value** |
| --- | --- | --- | --- | --- | --- | --- |
| All-cause mortality |  |  |  |  |  |  |
|  | Data source | NHANES vs other sources | -0.042 | 0.069 | -0.177 to 0.093 | 0.539 |
|  | Follow-up | ≥10 years vs <10 years | 0.065 | 0.065 | -0.063 to 0.193 | 0.320 |
|  | Age | <60 vs ≥60 years | 0.081 | 0.060 | -0.036 to 0.198 | 0.174 |
|  | Region | China vs the US. | -0.117 | 0.120 | -0.352 to 0.118 | 0.329 |
|  |  | Europe/other vs the US. | -0.020 | 0.085 | -0.187 to 0.147 | 0.813 |
|  | Population type | CVD vs general population | -0.009 | 0.091 | -0.187 to 0.169 | 0.920 |
|  |  | Stroke vs general population | 0.058 | 0.139 | -0.214 to 0.330 | 0.676 |
|  |  | Metabolic vs general population | 0.097 | 0.118 | -0.134 to 0.328 | 0.411 |
|  |  | Inflammatory vs general population | 0.071 | 0.110 | -0.145 to 0.287 | 0.519 |
|  |  | Respiratory vs general population | 0.063 | 0.094 | -0.121 to 0.247 | 0.503 |
|  |  | Diabetes vs general population | 0.066 | 0.075 | -0.081 to 0.213 | 0.379 |
|  |  | Other chronic diseases vs general population | -0.035 | 0.104 | -0.239 to 0.169 | 0.737 |
|  | Controlling for BMI | Yes vs No | 0.072 | 0.064 | -0.053 to 0.197 | 0.260 |
|  | Controlling for CVD | Yes vs No | -0.014 | 0.059 | -0.130 to 0.102 | 0.814 |
|  | Controlling for hypertension | Yes vs No | 0.030 | 0.067 | -0.101 to 0.161 | 0.654 |
|  | Sex, proportion | Male, %≥50% vs <50% | 0.036 | 0.061 | -0.084 to 0.156 | 0.556 |
|  | Controlling for diabetes | Yes vs no | -0.004 | 0.067 | -0.135 to 0.127 | 0.952 |
|  | Controlling for physical activity | Yes vs no | 0.035 | 0.066 | -0.094 to 0.164 | 0.594 |
| CV mortality |  |  |  |  |  |  |
|  | Data source | NHANES vs other source | 0.047 | 0.099 | -0.147 to 0.241 | 0.635 |
|  | Follow-up | <10 years vs ≥10 years | -0.010 | 0.084 | -0.176 to 0.155 | 0.902 |
|  | Age | <60 vs ≥60 | -0.002 | 0.097 | -0.192 to 0.188 | 0.984 |
|  | Region | The US. vs Europe/others | 0.043 | 0.105 | -0.163 to 0.249 | 0.682 |
|  |  | China vs Europe/others | -0.162 | 0.143 | -0.443 to 0.119 | 0.259 |
|  | Population type | CVD vs general population | -0.148 | 0.114 | -0.372 to 0.075 | 0.193 |
|  |  | Metabolic vs general population | 0.094 | 0.134 | -0.168 to 0.357 | 0.480 |
|  |  | Inflammatory vs general population | 0.042 | 0.169 | -0.289 to 0.374 | 0.802 |
|  |  | Respiratory vs general population | 0.334 | 0.153 | 0.034 to 0.634 | 0.029 |
|  |  | Diabetes vs general population | -0.043 | 0.145 | -0.327 to 0.241 | 0.766 |
|  |  | Other chronic disease vs general population | -0.047 | 0.184 | -0.407 to 0.313 | 0.797 |
|  | Controlling for BMI | Yes vs no | 0.006 | 0.091 | -0.172 to 0.185 | 0.945 |
|  | Controlling for CVD | Yes vs no | 0.129 | 0.096 | -0.058 to 0.317 | 0.176 |
|  | Controlling for hypertension | Yes vs no | 0.060 | 0.096 | -0.129 to 0.249 | 0.533 |
|  | Sex, proportion | Male, % ≥50% vs <50% | 0.001 | 0.096 | -0.187 to 0.189 | 0.989 |
|  | Controlling for diabetes | Yes vs no | 0.050 | 0.098 | -0.143 to 0.244 | 0.610 |
|  | Controlling for physical activity | Yes vs no | 0.021 | 0.097 | -0.169 to 0.212 | 0.826 |

# Appendix Table 6 **Predicted Hazard Ratios and 95% Confidence Intervals for All-Cause Mortality.**

| WWI (Dose) | Mean dose (Spline) | Predicted HR | 95%CI |
| --- | --- | --- | --- |
| 8.2 | 8.66 | 1 | 1 |
| 9.2 | 9.92 | 1.34 | 1.15-1.56 |
| 10.2 | 11.17 | 1.79 | 1.32-2.44 |
| 11.2 | 12.42 | 2.41 | 1.52-3.81 |
| 12.2 | 13.68 | 3.23 | 1.75-5.96 |
| 13.2 | 14.93 | 4.33 | 2.01-9.31 |
| 14.2 | 16.19 | 5.81 | 2.32-14.55 |

# Appendix Table 7 **Predicted Hazard Ratios and 95% Confidence Intervals for CV Mortality.**

| WWI (Dose) | Mean dose (Spline) | Predicted HR | 95%CI |
| --- | --- | --- | --- |
| 8.2 | 8.56 | 1 | 1 |
| 9.2 | 9.80 | 1.18 | 1.09-1.29 |
| 10.2 | 11.04 | 1.41 | 1.18-1.68 |
| 11.2 | 12.28 | 1.68 | 1.29-2.18 |
| 12.2 | 13.53 | 2.00 | 1.41-2.83 |
| 13.2 | 14.77 | 2.38 | 1.53-3.68 |
| 14.2 | 16.01 | 2.83 | 1.67-4.78 |

# **References**

1. Zhang Y, Yao Y. The association between obesity indicators and mortality among individuals with hyperlipidemia: evidence from the NHANES 2003–2018*.* Lipids in Health and Disease, 2025;24: 20.

2. Zierfuss B, Hbaus C, Herz C T, Pesau G, Schernthaner G H. Predictive power of novel and established obesity indices for outcome in PAD during a five-year follow-up*.* Nutrition Metabolism and Cardiovascular Diseases, 2020;30: 1179-1187.

3. Li H, Zhong W, Cheng H, Wang S, Li R, Wang L, et al. Association between weight-adjusted-waist index and long-term prognostic outcomes in cardiovascular disease patients: results from the NHANES 1999–2018 study*.* Diabetology & Metabolic Syndrome, 2025;17: 1-15.

4. Zhou T, Wu J, Wang Y, Gao Y, Cheng K. Weight-Adjusted Waist Index , Psoriasis , and All-Cause Mortality : Findings from the NHANES 2003 - 2006 and 2009 - 2014*.* CLINICAL COSMETIC AND INVESTIGATIONAL DERMATOLOGY, 2025: 7-18.

5. Liu F, Chen J, Yao Y, Ren R, Yu Y, Hu Y. Sex-specific association of weight-adjusted waist index with mortality in stroke survivors : A national longitudinal cohort study*.* NUTRITION METABOLISM AND CARDIOVASCULAR DISEASES, 2025;35: 103743.

6. Li S, Jin J, Zhang W, Cao Y, Qin H, Wang J, et al. Association of weight-adjusted waist index with all-cause and cause-specific mortality among cancer survivors : a cohort study of the NHANES 1999-2018*.* FRONTIERS IN ENDOCRINOLOGY, 2024: 1422071.

7. Liu W, Yang X, Zhan T, Huang M, Tian X, Tian X, et al. Weight-adjusted waist index is positively and linearly associated with all-cause and cardiovascular mortality in metabolic dysfunction-associated steatotic liver disease : findings from NHANES 1999-2018*.* FRONTIERS IN ENDOCRINOLOGY, 2024: 1457869.

8. Liu S, Yu J, Wang L, Zhang X, Wang F, Zhu Y. Weight-adjusted waist index as a practical predictor for diabetes , cardiovascular disease , and non-accidental mortality risk*.* NUTRITION METABOLISM AND CARDIOVASCULAR DISEASES, 2024;34: 2498-2510.

9. Cai S, Zhou L, Zhang Y, Cheng B, Zhang A, Sun J, et al. Association of the Weight-Adjusted-Waist Index With Risk of All-Cause Mortality : A 10-Year Follow-Up Study*.* FRONTIERS IN NUTRITION, 2022: 894686.

10. Tao Z, Zuo P, Ma G. The association between weight-adjusted waist circumference index and cardiovascular disease and mortality in patients with diabetes*.* SCIENTIFIC REPORTS, 2024: 18973.

11. Wang S, Li D, Sun L. Weight-adjusted waist index is an independent predictor of all-cause and cause-specific mortality in patients with asthma*.* HEART & LUNG, 2024: 166-174.

12. Ding C, Shi Y, Li J, Li M, Hu L, Rao J, et al. Association of weight-adjusted-waist index with all-cause and cardiovascular mortality in China : A prospective cohort study*.* NUTRITION METABOLISM AND CARDIOVASCULAR DISEASES, 2022;32: 1210-1217.

13. Tao Z, Zuo P, Ma G. Association of weight-adjusted waist index with cardiovascular disease and mortality among metabolic syndrome population*.* SCIENTIFIC REPORTS, 2024: 18684.

14. Han Y, Shi J, Gao P, Zhang L, Niu X, Fu N. The weight-adjusted-waist index predicts all-cause and cardiovascular mortality in general US adults*.* CLINICS, 2023: 100248.

15. Zhao P, Du T, Zhou Q, Wang Y. Association of weight-adjusted-waist index with all-cause and cardiovascular mortality in individuals with diabetes or prediabetes : a cohort study from NHANES 2005 - 2018*.* SCIENTIFIC REPORTS, 2024: 24061.

16. Cao T, Xie R, Wang J, Xiao M, Wu H, Liu X, et al. Association of weight-adjusted waist index with all-cause mortality among non-Asian individuals : a national population-based cohort study*.* NUTRITION JOURNAL, 2024: 62.

17. Zhang T, Zhang Z, Wang X, Kuang H, Xu Q, Li H, et al. Relationship between weight-adjusted-waist index and all-cause and cardiovascular mortality in individuals with type 2 diabetes*.* DIABETES OBESITY & METABOLISM, 2024;26: 5621-5629.

18. Jiao X, Huang L, Lin L, Zhu F, Fan F, Xiao J, et al. Association of weight-adjusted waist index with preserved ratio impaired spirometry and all-cause mortality*.* Frontiers in nutrition, 2025;12: 1594453.

19. Peng M, Zhang X. Association between weight-adjusted waist index and mortality: The mediative role of depressive symptoms*.* Journal of affective disorders, 2025;386: 119434.

20. Liu S, Chen S, Huang Y, Man Q, Yang Y, Wen J, et al. Association of weight-adjusted waist index with all-cause and cardiovascular disease mortality among rheumatoid arthritis population: a cohort study from the NHANES 1999-2018*.* Lipids in health and disease, 2025;24: 223.

21. Lan Y, Zheng Q, Lin C, Lin K, Chen Y. Association of weight-adjusted-waist index with all-cause and cardiovascular mortality in hypertension: a prospective cohort study*.* BMC public health, 2025;25: 2628.

22. Guo S, Chen D, Zhang Y, Cao K, Xia Y, Yang D. Association of weight-adjusted waist index with all-cause and cardiovascular mortality in individuals with osteoarthritis*.* BMC musculoskeletal disorders, 2025;26: 390.

23. Chen J, Zeng X, Xiang N, Luo R, Xiao Z, Chen R, et al. The L-shaped association between weight-adjusted-waist index and all-cause mortality in individuals with psoriasis: results from NHANES database retrospective cohort study*.* Frontiers in immunology, 2025;16: 1548788.

24. Pan L, Xu X, Zhang M, Sun C, Xu W. Weight-adjusted waist index and mortality in diabetic retinopathy: a NHANES 1999-2018 cohort study*.* Diabetology & metabolic syndrome, 2025;17: 355.

25. Zhang X, Zhang W, Hu Y, Yang G, Liu S, Hao W, et al. Association of Weight-Adjusted Waist Index with All-Cause and Cardiovascular Mortality in Patients with Chronic Obstructive Pulmonary Disease: A Retrospective Cohort Study*.* International journal of chronic obstructive pulmonary disease, 2025;20: 3647-3658.

26. Zheng Y, Nie Z, Zhang Y, Sun T. The weight-adjusted-waist index predicts all-cause and cardiovascular mortality in hypertension*.* Frontiers in cardiovascular medicine, 2025;12: 1501551.

27. Li H, Zhong W, Cheng H, Wang S, Li R, Wang L, et al. Association between weight-adjusted-waist index and long-term prognostic outcomes in cardiovascular disease patients: results from the NHANES 1999-2018 study*.* Diabetology & metabolic syndrome, 2025;17: 19.

28. Lyu Q, Ma L, Liu H, Shao H. Association of the weight-adjusted waist index with mortality in osteoporotic patients*.* Journal of bone and mineral metabolism, 2025;43: 384-391.

29. Yeo Y H, Zhu Y, Gao J, Liu S, Ni W, Rui F, et al. Anthropometric Measures and Mortality Risk in Individuals With Metabolic Dysfunction-Associated Steatotic Liver Disease (MASLD): A Population-Based Cohort Study*.* Alimentary pharmacology & therapeutics, 2025;62: 168-179.

30. Ren K, Tao Y, Wang M. Association between physical activity, weight - adjusted waist index, and all - cause mortality in Chinese older adults: a national community - based cohort study*.* Biology of sport, 2025;42: 323-331.

31. Guo F, Guo C, Dou J, Wang J, Wu R, Song S, et al. Association of surrogate adiposity markers with prevalence, all-cause mortality and long-term survival of heart failure: a retrospective study from NHANES database*.* Frontiers in endocrinology, 2025;16: 1430277.

32. Wang H, Cai W, Zeng H, Xu Z, Luo X, Wu J, et al. Inflammatory markers mediate the association between weight-adjusted waist circumference and mortality in patients with cardiometabolic syndrome*.* Scientific reports, 2025;15: 8505.

33. Hu J, Tang S, Zhu Q, Liao H. Predictive value of six anthropometric indicators for prevalence and mortality of obstructive sleep apnoea asthma and COPD using NHANES data*.* Scientific reports, 2025;15: 16190.

34. Liu M, Pei J, Zeng C, Xin Y, Tang P, Hu X. Association and predictive value of cumulative weight-adjusted waist index with all-cause mortality in type 2 diabetes: Insights from the ACCORD study*.* Nutrition, metabolism, and cardiovascular diseases : NMCD, 2026;36: 104416.

35. Wang K, Li Y, Chen Y. Anthropometric indices and mortality in postmenopausal women: NHANES 1999-2018 evidence*.* BMC women's health, 2025;25: 623.

36. Fan B, Ren K, Li L. The joint effect of weight-adjusted waist index and physical activity on all-cause mortality in Chinese elderly patients with multimorbidity: A study based on the CLHLS from 2011 to 2018*.* PloS one, 2025;20: e325886.

37. Wu C, Pan C, Liu L, Li W. Association between weight-adjusted waist index and risk of mortality and disease progression in participants with chronic kidney disease: a prospective study from the UK Biobank*.* Frontiers in nutrition, 2026;13: 1646414.

38. Soler-Espejo E, Chen Y, Roldán V, Marín F, Rivera-Caravaca J M, Lip G Y. Prognostic value of adiposity indices in anticoagulated patients with atrial fibrillation: the prospective Murcia AF Project III cohort*.* The American journal of clinical nutrition, 2026;123: 101184.

39. Huang Y, Zhang X, Zhu H, Zhang M. The joint association between inflammation and centripedal obesity with mortality risk in patients with preserved ratio impaired spirometry*.* NPJ primary care respiratory medicine, 2025;35: 50.

40. Xia X, Chen S, Tian X, Xu Q, Zhang Y, Zhang X, et al. Roles of general and central adiposity in cardiometabolic multimorbidity: revisiting the obesity paradox using a multistate model*.* Obesity (Silver Spring, Md.), 2024;32: 810-821.

41. Sun C H, Mao F, Zhang R, You X Q, Li J H. Association of weight-adjusted waist circumference index and its change with risk for cardiovascular and cerebrovascular disease mortality: a cohort study*.* Zhonghua liu xing bing xue za zhi = Zhonghua liuxingbingxue zazhi, 2025;46: 1919-1927.

42. Li J, Liang W, Song J, Zhang K, Gong M, Mang J. Beyond BMI: Finding critical body adiposity indices for dual-threat prediction of depression and mortality in stroke survivors*.* Journal of affective disorders, 2026;394: 120564.

43. Zhang W, Yang G, Hu Y, Zhang X, Liu S, Zhu X, et al. Correlation between two novel visceral fat indicators and all-cause mortality among patients with asthma*.* BMC pulmonary medicine, 2026;26: 17.

44. Liu W, Chen R, Li Q, Zhu Y, Jin Y, Zhao K, et al. Prognostic value of abdominal obesity indicators for all-cause mortality in familial hypercholesterolemia*.* Clinical nutrition ESPEN, 2026;72: 102953.

45. Moshkovits Y, Chetrit A, Dankner R. Association of body fat-distribution markers with ischemic ECG changes and with 20-year all-cause and cardiovascular mortality in community-dwelling older adults*.* Postgraduate medical journal, 2026: qgaf190.

46. Zhang Z, Zhou L, Xu Y, Yao L, Ma T, Pan X. The impact of obesity-related anthropometric indices on diabetic foot ulcer and mortality: analysis of a nationally representative sample*.* Hormones (Athens, Greece), 2025: 10-1007.

47. Zhang Q, Wang Y, Liu S, Zhu S, Li P, Zhang S, et al. Mortality risk associated with general and central obesity in inflammatory bowel disease patients: a long-term prospective cohort study*.* International journal of obesity (2005), 2025;49: 2303-2310.
